# Recent Development of Photochromic Polymer Systems: Mechanism, Materials, and Applications

**DOI:** 10.34133/research.0392

**Published:** 2024-06-17

**Authors:** Jindou Zou, Jimeng Liao, Yunfei He, Tiantian Zhang, Yuxin Xiao, Hailan Wang, Mingyao Shen, Tao Yu, Wei Huang

**Affiliations:** ^1^ Frontiers Science Center for Flexible Electronics (FSCFE) and Xi’an Institute of Flexible Electronics (IFE), Northwestern Polytechnical University, Xi’an 710072, China.; ^2^ Key Laboratory of Flexible Electronics of Zhejiang Province, Ningbo Institute of Northwestern Polytechnical University, Ningbo 315103, China.; ^3^ Key Laboratory of Flexible Electronics (KLOFE) and Institute of Advanced Materials (IAM), Nanjing Tech University (Nanjing Tech), Nanjing 211816, China.; ^4^ State Key Laboratory of Organic Electronics and Information Displays and Jiangsu Key Laboratory of Biosensors, Institute of Advanced Materials (IAM), Nanjing University of Posts and Telecommunications, Nanjing 210023, China.

## Abstract

Photochromic polymer is defined as a series of materials based on photochromic units in polymer chains, which produces reversible color changes under irradiation with a particular wavelength. Currently, as the research progresses, it shows increasing potential applications in various fields, such as anti-counterfeiting, information storage, super-resolution imaging, and logic gates. However, there is a paucity of published reviews on the topic of photochromic polymers. Herein, this review discusses and summarizes the research progress and prospects of such materials, mainly summarizing the basic mechanisms, classification, and applications of azobenzene, spiropyran, and diarylethene photochromic polymers. Moreover, 3-dimensional (3D) printable photochromic polymers are worthy to be summarized specifically because of its innovative approach for practical application; meanwhile, the developing 3D printing technology has shown increasing potential opportunities for better applications. Finally, the current challenges and future directions of photochromic polymer materials are summarized.

## Introduction

Photochromic polymers are polymers containing photochromic groups in the macromolecules, including polymers produced by physical doping and chemical polymerization methods [1,2]. Photochromic materials can be classified by their nature into organic photochromic materials [3,4], inorganic photochromic materials, and organic–inorganic hybrid photochromic materials. For inorganic photochromic compounds, there are 3 main categories: the first is transition metal oxides that undergo reversible redox [5] such as WO_3_, MoO_3_, TiO_2_, etc. The second category is metal halides [6], whose color-changing mechanism is based on the change in valence of metal ions [7]. For example, Ce-doped calcium fluoride crystals produce lattice defects that turn the colorless Ce^3+^ into pink defects [8]. The third category is rare earth complexes, such as Dy, Eu, Tb, etc, which are due to their photogenerated radicals inducing photochromism [9].

**Fig. 1.**
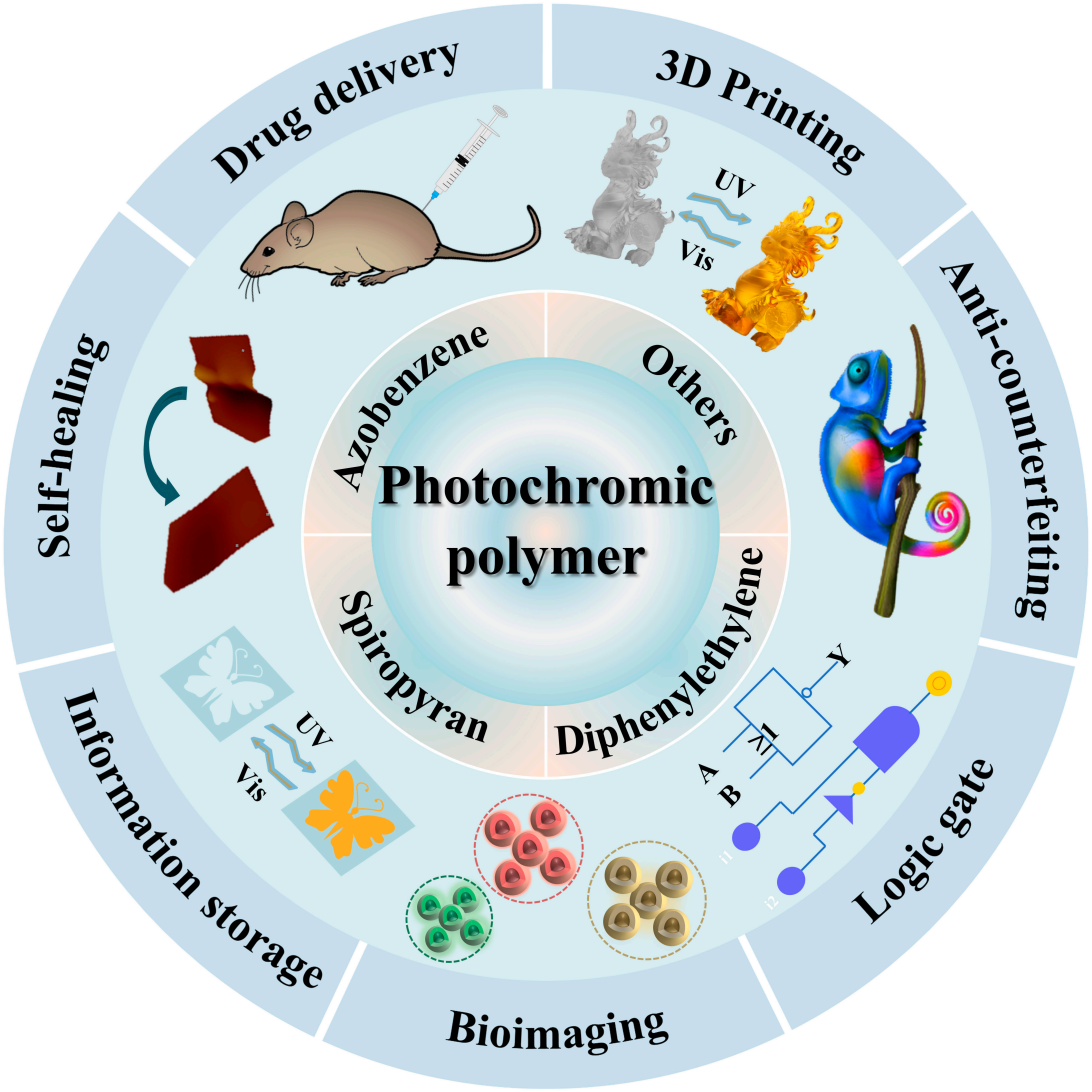
Classification and applications of photochromic polymers.

For organic photochromic materials [10], they can be divided into 6 categories [11–13] according to the reaction mechanisms: the heterogeneous cleavage of spiropyran (SP) and spirooxazine bonds [14], the homogeneous cleavage of hexaphenyl-bis-imidazole bonds, the proton transfer reciprocal isomerization of salicylaldehyde condensed aniline compounds, the *cis*-*trans* isomerization of azobenzenes [15–17], the redox of thick-ringed aromatic compounds as well as the pericyclic reaction systems of captive arginine anhydride and heterocyclic diarylethene (DAE) groups [18,19]. On the other hand, photochromic polymers attract much attention for their rapid and convenient response as photostimuli reversibility of response [20], controllability, accessibility, and nondestructive properties [21]. They also have shown their promising performances in a series of applications, such as photo actuators [22], photolithographic photopatterning [23], drug delivery [24], and molecular switches [25]. Methods to prepare photochromic polymers include blending organic photochromic molecules with polymers [26], introducing side chains, introducing main chains, etc [27]. In this review, several classes of common photochromic polymers, namely azobenzene, SP, DAE, and other photochromic polymers, will be overviewed, and their related applications and classifications will be summarized. Current developments and challenges of combining photochromic polymers will also be discussed (Fig. 1). Due to the limitation of scope, this review could not include all achievements related to this research area, and only a selection of representative examples will be introduced. Readers are encouraged to refer to other excellent review papers in the literature for additional examples [28–30], like photoresponsive smart materials [31], nonlinear photochromic materials [32], 3-dimensional (3D) printing of photochromic liquid crystal elastomers [33], photochromic photoinitiators [34], 3D printing of photochromic hydrogels [35] and photochromic optical control devices [36].

## Azobenzene

### Overview

Azobenzenes and their derivatives are an important part of the family of photochromic compounds. Azobenzene compounds are typical compounds containing N=N bonds and they can undergo *cis*-*trans* isomerizations upon ultraviolet (UV) and visible light irradiation. The forms and strengths of the absorption band in *trans*-isomers under different irradiation differ obviously, manifested as a stronger π-π* band in the UV region and a weaker n-π* band in the visible light region. However, after a *trans*-isomer is transformed to a *cis*-isomer, the *cis*-isomer exhibits a stronger n-π* band in the visible range, and the existing visible light can induce the reversible process correspondingly. Also, the *cis*-isomer can revert to the *trans*-isomer upon thermal relaxation, as the latter has a higher thermal stability [37].

There are 2 main mechanisms for the photoisomerization of azobenzene. One of the mechanisms is that the N=N bond takes on the nature of a single bond after excitation and then rotates to achieve isomerization; the other is that isomerization is achieved through the inversion of C–N bond. In recent years, there have been extensive reports on the synthesis and classification of azobenzene derivatives. The characteristics of easy synthesis and high fatigue resistance make azobenzene polymers an important class of photoresponsive material optical switches. In 1997, the photoisomerization activity of an azobenzene core polymer was first reported [38]. After that, there have been many studies on the materials and applications of azobenzene derivatives.

### Classification of azobenzene polymers

#### Photoresponsive block polymers

**Fig. 2.**
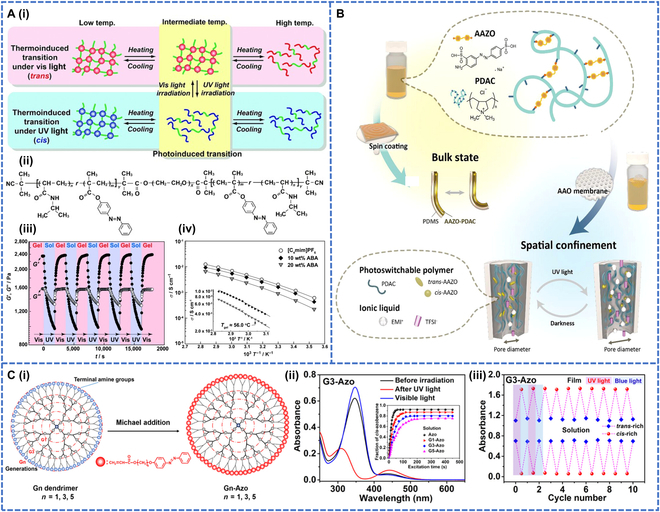
(A) Properties of ABA block polymers: (A-i) schematic representation of photoreversible ion gels, (A-ii) chemical structure of the ABA triblock copolymer, (A-iii) reversible sol–gel transition cycle of the ABA triblock copolymer, and (A-iv) relationship between ionic conductivity of ABA triblock copolymer ion gels near the sol–gel transition temperature. Reproduced with permission from [40]. Copyright 2015, American Chemical Society. (B) Graphical illustrations of the photoswitchable polymer system, in which the photomechanical properties and on-demand ionic conductivity can be achieved via light irradiations. Reproduced with permission from [41]. Copyright 2023, John Wiley & Sons Inc. (C-i) Schematic diagram of synthesis of photochromic dendrimers. (C-ii) UV–vis absorption spectra of G3-Azo in tetrahydrofuran before irradiation and after UV irradiation. (C-iii) Cycling performances of G3-Azo in solution and the film under the alternative UV light and visible light irradiation. Reproduced with permission from [47]. Copyright 2020, American Chemical Society.

Azobenzene polymers are divided into block copolymers (BCs) and dendrimers in this paper. A BC is a polymer consisting of 2 or more polymers connected together by chemical binding. When there is an azobenzene photoresponsive group in the polymer molecule, the polymer becomes a photoresponsive BC [39]. An ABA triblock polymer system whose side chain had an azobenzene moiety was synthesized [40]. Block A consisted of a random copolymer of thermosensitive *N*-isopropylacrylamide (NIPAm) units and azobenzene-containing methacrylate units. Block B consisted of poly(ethylene oxide) (PEO) units that were soluble in ionic liquids (Fig. 2A-i and ii). The polymer had a sol–gel transition and was reversible at least 5 times (Fig. 2A-iii), changing from *trans* to *cis* in the dark state under UV irradiation at 366 nm. This changed the dipole moment and disrupted the micellar structure. Under visible light irradiation at 440 nm, this process was reversed, the *trans* state was restored, and the micelles were reorganized. This photoreversible ionogel showed high ionic conductivity and was also important for processing ionic liquid materials to develop solid films and nanopatterning applications (Fig. 2A-iv). The azobenzene cross-linked polymer (AAZO-PDAC) was used to create photoswitchable ion channel designs inspired by guard cells [41]. Under UV irradiation, *trans* azobenzene was converted to *cis*, compressing the volume of the polymer network; conversely, the structure was restored in the dark. The polymer was doped with polydimethylsiloxane to obtain the photomechanically deformable film AAZO-PDAC/PDMS, and the film deformation was found to be stable after 20 cycles. This polymer with photoswitching and photomechanical effects was expected to be used as a sensor and in the biomedical field (Fig. 2B).

According to the structural changes induced by light, Zhao [42] created a new classification that includes 4 types of BC micelles. The structural changes of most of the photochromic BC were associated to a change in the equilibrium level of hydrophobicity and hydrophilicity. The photochromic part of the side group, acting as a hydrophobic block, was bound to the BC structure. When the micellar solution was exposed to light, the photoreaction could be designed to increase the polarity of the hydrophobic block or to convert it into a hydrophilic block. The block junctions of the BC could also be disrupted to disconnect the hydrophilic and hydrophobic blocks by light. Another type of photochromic BC involved the repeated insertion of the photodissociable part of the backbone into the core of the hydrophobic micelle to form a block so that degradation could be readily induced upon photoirradiation. The last type of photochromic BC made use of reversible cross-linking for the stabilization of the BC micelle by first cross-linking it with light at a certain wavelength, and the cross-link could be subsequently destabilized by light at a different wavelength.

Although light-responsive block polymers can undergo photoisomerization under light, their poor photostability, slow response, high dependence on the environment, and short lifespan make the application still have many limitations and need to be carefully considered in specific applications.

#### Dendritic polymers

Dendritic polymers are a class of polymers with 3D structures, so called because of the tree-like structures of their backbone with many ends [43]. Dendritic polymers are one of the most highly branched types of polymers having drawn much attention from scientists in many fields [44] due to their aesthetics and high symmetry of structures, extremely high functional group density on the surface, controllability of parameters such as molecular size and shape, as well as the uniqueness of their properties [45]. Typical dendritic macromolecules exhibit a visually spherical morphology, with a high density of functional groups on the surface, a tight exterior, a loose interior, and adjustable internal cavities. Since 1993, when the first dendrimer with a benzene ring as the center and an azobenzene-based periphery was prepared by “emanation”, the study of dendrimers containing azobenzene photoresponsive groups had quickly become a hot topic. The first examples of dendrimers that could adjust their sizes in solution upon light irradiation were reported [46]. Irradiation with 350-nm light led to the partial isomerization of azobenzene to the *cis*-form while the reversible process could occur under the visible light. Xu et al. [47] synthesized an azobenzene-containing dendrimer with excellent solid–liquid reversible transition and solar thermal conversion properties by Michael addition reactions between 3 different generations of poly (G1, G3, and G5) dendrimers and azobenzene acrylate (Fig. 2C-i). The spectrum of polymer G3 before and after UV absorption was shown, and G3 can be recycled at least 10 times (Fig. 2C-ii and iii). The photochromic dendrimers could be used as adhesives to realize healable coatings, in which the adhesives were switchable under UV and visible light, and could effectively repair scratches on dendritic macromolecule coatings. Moreover, the bond strengths and the solar energy storage densities of the photochromic macromolecule fuel increased markedly with the number of dendrimer generations.

In addition, the photocontrol protein nanowires with reversible morphologies were constructed by photoisomerization of dendrimers inducing self-assembly of the SP1 protein [48]. UV irradiation of polymers solution for 10 min reduced its absorption intensity to 0.520 at 367 nm (1.66), indicating that polymer was isomerized to the *cis*-form. *Cis*-polymers could manipulate the tilted alignment of SP1 to produce protein nano-arc. Upon visible light irradiation for 4 min, it could completely return to the original state, and the process was fully reversible and reproducible.

Although the branching structure of azobenzene dendritic polymers gives them versatility, synthesizing these polymers and controlling the molecular structure are difficult, which limits their application areas, requiring careful consideration and trade-offs in specific applications.

### Azobenzene polymer applications

#### Photoactivation and photopatterning

**Fig. 3.**
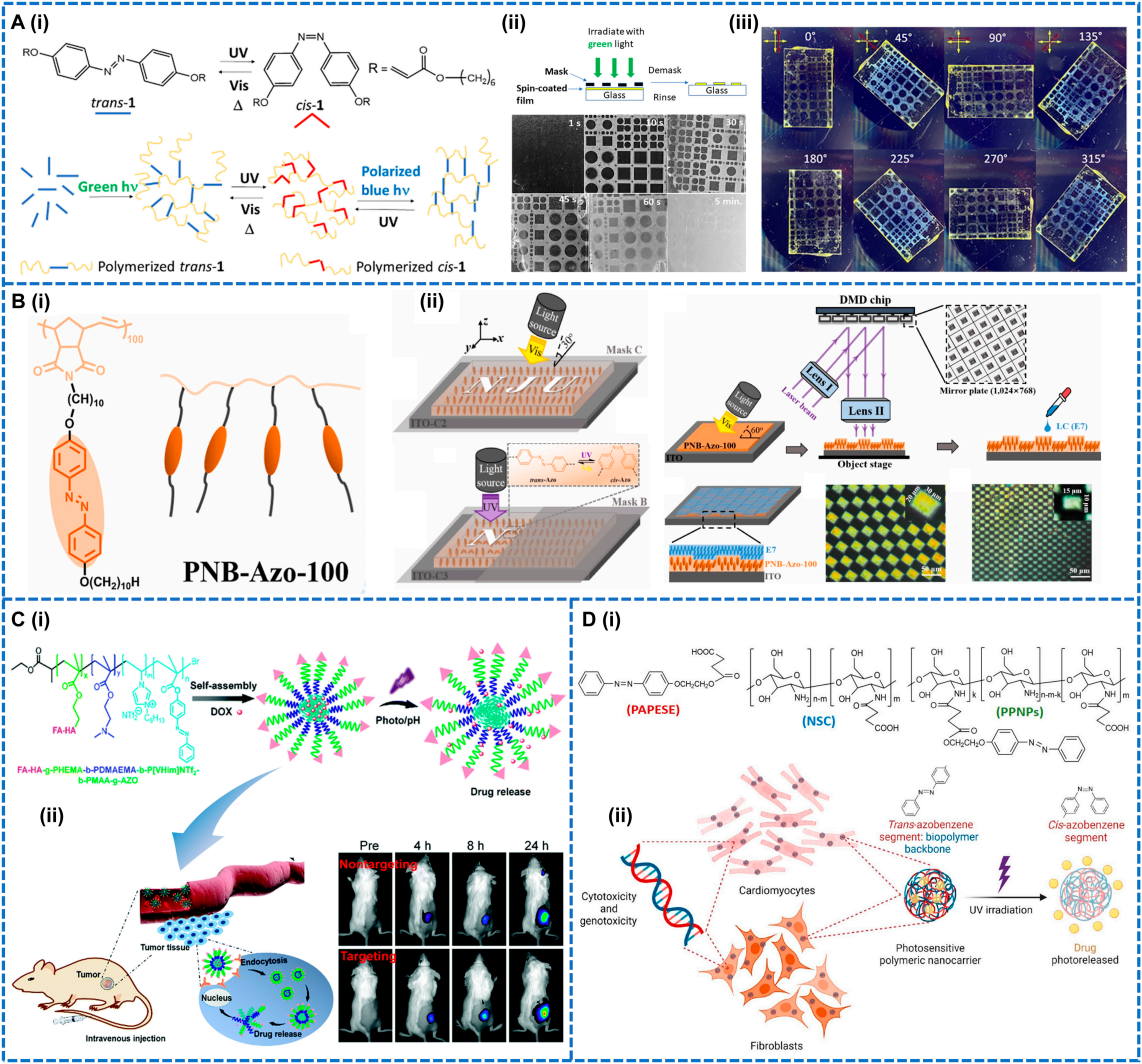
(A-i) Schematic description of *trans*-*cis* isomerization (Azo-LC), polymerization, cross-linking, writing, and erasing procedures. (A-ii) Spin-coated films were masked and irradiated with monochromatic green light (532 nm). (A-iii) Birefringence pattern formed when UV light irradiates the film. Reproduced with permission from [52]. Copyright 2023, American Chemical Society. (B-i) Chemical structures of PNB-Azo-100. (B-ii) Application of azobenzene light patterning. Reproduced with permission from [53]. Copyright 2021, Elsevier Ltd. (C-i) Structure of polymer NPs. (C-ii) Polymer NPs for cancer therapy. Reproduced with permission from [58]. Copyright 2020, Royal Society of Chemistry. (D-i) Chemical structures of PPNPs, PAPESE, and *N*-succinyl chitosan. (D-ii) Schematic illustration of cytotoxicity and genotoxicity of azobenzene-based polymeric nanocarriers for phototriggered drug delivery in biomedical applications. Reproduced with permission from [61]. Copyright 2022, Licensee MDPI.

Azobenzene photochromic polymers achieved important progress in the domain of photopatterning applications [49], where they found valuable uses in crafting photosensitive security labels [50] and cards that greatly deterred counterfeiting efforts and ensured information security [51]. Carroll et al. [52] reported an azobenzene liquid crystal polymer (Azo-LC) material for photopatterning, which was synthesized by mixing azobenzene diacrylate with triethylamine as the catalyst and heating at 65 °C for 1 h to prepare azobenzene cross-linked films. The photopolymerized films were irradiated with UV and visible light so that the individual azobenzene molecule structures could switch between the *trans*- and *cis*-isomers (Fig. 3A-i). Irradiation of the *cis*-isomer with linearly polarized blue light produced a birefringent single-domain oriented film, forming the *trans* chromophore of the liquid crystal structure. UV irradiation eliminated the liquid crystal state, which could be regenerated under linearly polarized blue light. The photomask directed green light irradiation to produce cured and patterned films. Covered with a quartz photomask and exposed to green light at different times, the green light initiated a polymerization reaction in the transparent region of the photomask to form an insoluble network of azobenzene repeating units. After green light exposure, the unpolymerized areas were removed by rinsing with toluene to obtain a visually recognizable pattern on the substrate. The light patterns were exposed for 10 to 60 s, and the results showed that the patterns were sharpest and cleanest at 10 to 45 s (Fig. 3A-ii). In addition, birefringent patterns could be formed with this polymer film (Fig. 3A-iii).

Photopatterned films were also utilized for high-resolution imaging, which boasted advantages such as high sensitivity, real-time capabilities, and nondestructive detection. Photochromic polymers were renowned for their rapid light response rate, stable photogenerated colors, and notable and reversible color changes. These changes could be precisely captured by high-resolution imaging systems, enabling the generation of high-quality, high-definition photochromic patterns. Consequently, photopatterned high-resolution films were capable of accurately documenting the color change process and revealing their microstructural and performance characteristics, which had been reported in the past. Chen et al. [53] prepared side chain liquid crystalline azo polymers with different dissipative particles of poly-norbornene (PNB) backbone by ring-opening metathesis polymerization and observed excellent photoresponsiveness (Fig. 3B-i). The PNB-Azo-100 film could be used as an optical orientation modulation command surface for the nematic liquid crystal E7, allowing macroscopic graphics to be written through a photomask. In addition, the PNB-Azo-100 film enabled tessellation patterns with a grid resolution of up to 100 μm. The film also exhibited sufficient chemical stability and structural reproducibility for potential applications in anti-counterfeiting and smart technology (Fig. 3B-ii).

To explore the light-driven patterning properties of azobenzene polymers, Li et al. [54] studied a linear polyurea containing a bridged azobenzene part in the main chain (PbAzo) by an addition polymerization reaction. The *cis*-*trans* isomerization occurred under 405-nm irradiation, accompanied by a reversible transition of color from yellow to red. Irradiation at 532 nm or heating caused *trans*-to-*cis* changes in PbAzo for its better thermal stability in *cis*-form. The good photochromic properties of PbAzo made it a rewriteable material for optical patterning. Patterns could be written under 405-nm irradiation and erased by irradiation at 532 nm or by heating. In addition, PbAzo could be used for optical drivers. The PbAzo film could be bent under 405-nm irradiation, and the bending would be restored at 532-nm irradiation. This light-driven oscillation of the PbAzo film could cycle more than 30 times with little attenuation in the bending angle or the bending speed.

#### Drug delivery

Introducing azobenzene photosensitive compounds into polymeric assemblies allows the precise and remote control of the structural and photochromic properties [55] under illumination with light of a certain wavelength [56]. Photoisomerization of azobenzene holds great promise in the field of biopharmaceuticals, where azobenzene polymers can be used to control the solubility or structure of the polymer through light-induced structural changes. This property can be used to design photosensitive drug delivery systems for controlled drug release [57]. Lu et al. [58] synthesized an amphiphilic BC comprising a targeting ligand (folic acid and hyaluronic acid), a light-responsive block (p-hydroxy azobenzene, AZO) and a pH-responsive block (Fig. 3C-i and ii). The polymers formed spherical nanoparticles (NPs) in water and demonstrated dual-responsive drug release capabilities by achieving controlled drug release in weakly acidic environments and under UV irradiation. These NPs were biocompatible and effective in inhibiting tumor growth, making them suitable for drug-controlled release materials.

In recent years, azobenzene polymers have been widely used as NPs [59]. Still, few studies have been made on the accurate assessment of the genotoxicity of azobenzene compounds [60]. Londoño-Berrío el al. [61] used the amide reaction between azobenzene derivate (PAPESE) and *N*-succinyl chitosan to synthesize photosensitive polymers and used nanoprecipitation methods for the self-assembly of polymeric photoresponse NPs (PPNPs) to prepare polymeric NPs (Fig. 3D-i). Under UV radiation, the azobenzene in PPNPs was capable of *trans*-*cis* photoisomerization, which could be utilized for drug delivery. This study represented the initial evaluation of the genotoxicity of azobenzene polymer NPs using biocompatibility and other analyses. The findings revealed that PPNPs had substantial potential in numerous medical applications, such as drug delivery, cellular diagnostic techniques, and photodynamic therapy (Fig. 3D-ii).

#### Self-healing

**Fig. 4.**
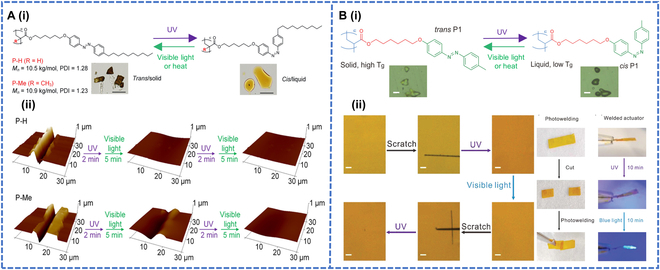
(A-i) Chemical structures and photoisomerization of azobenzene-containing polyacrylate P-H and polymethacrylate P-Me. (A-ii) AFM height images of P-H and P-Me films with a scratch before irradiation, after UV irradiation (365 nm, 12.8 mW cm^–2^), and after subsequent visible light irradiation (530 nm, 19.9 mW cm^–2^) for 2 cycles. Reproduced with permission from [62]. Copyright 2023, American Chemical Society. (B-i) Chemical structure and photoresponsive properties of the azopolymer P1. (B-ii) Reversible solid–liquid transition healing through light-induced transformation. Reproduced with permission from [63]. Copyright 2020, John Wiley & Sons Inc.

As mentioned earlier, certain azobenzene polymers have been widely used in healable materials due to their ability to switch their glass *T*_g_ under light irradiation conditions and exhibit photoinduced reversible solid–liquid transitions. Liang et al. [62] reported 2 polymers (P-H and P-Me), polyacrylate and polymethacrylate, which were modified with azobenzene (Fig. 4A-i). The former was found to show a quicker photoinduced reversible solid–liquid transition and could be used to make a fast-healing coating. P-H and P-Me coatings with the same depth and width of scratches were prepared by irradiating the coatings with UV light for 2 min, followed by visible light for 5 min. Under light irradiation, a reversible solid–liquid transition occurred, which induced the polymer chains to flow through the scratches. After light irradiation, the P-H coating healed completely. In contrast, the P-Me coating healed more slowly (Fig. 4A-ii).

Although azo polymers of polyacrylates have been reported several times in the literature, photoinduced reversible solid–liquid transitions of entangled high-molecular-weight azo polymers have been rarely investigated, nor have the fabrication of actuators by photoinduced reversible solid–liquid transitions been reported. Therefore, Chen et al. [63] attempted to use entangled linear azo polymers to prepare healable and processable photo actuators that could be repaired and reprocessed using solution treatment or light irradiation at an ambient temperature (Fig. 4B-i). The scratch on the P1-5k (azo-actuator) was partially irradiated with UV light for 20 min. The scratch disappeared as the UV light irradiation induced the flow of the azo polymer, after which visible light was used to switch the healed P1 back to the solid state to complete the healing process, and the photoinduced solid–liquid transition had facilitated the repair of the damaged actuator (Fig. 4B-ii).

Overall, the applications of azobenzene polymers are not limited to the ones we have mentioned here, and the development of more attractive applications is still a major focus of researchers, hopefully for aerospace and wearable technology in the future.

## Spiropyran

### Overview

SPs are among the earliest systems of organic photochromic materials that have been reported. They consist of 2 heteroaromatic rings (one of which is a pyran ring) structurally linked by sp^3^ hybridized spiro atoms, with the 2 ring systems orthogonal to each other and not conjugated. At that time, the compound was irradiated with UV light to form a phycocyanin-like structure, which could be restored to its original state when heated or exposed to visible light after the removal of the excitation source. Although SP exhibits poor fatigue resistance, it is multiresponsive in terms of light [64], heat [65], pH [66], and electricity. Hence, SP and its derivatives and polymers have been widely used in recent years.

The absorption of SP occurs in the UV spectral region, generally in the range of 200 to 400 nm, and the compounds are colorless. Upon UV excitation, the C–O bond in the molecule undergoes isomerization, followed by isomerization and rearrangement of the molecular structure and electronic configuration, with the changing of 2 ring systems from an orthogonal arrangement to a coplanar structure such that the whole molecule forms a large conjugated system. This is accompanied by a corresponding red shift in the absorption spectrum, which occurs in the 500- to 600-nm range, causing the compound to become colored. This molecule after ring opening is usually referred to merocyanine (MC). The ring closure reaction of MC back to SP occurs under visible light or heat, constituting a photochromic system with a reversible photochromic process. In addition to solutions, SP can exhibit photochromic properties in resins and polymers.

### Classification of SP polymers

#### Polymer film

**Fig. 5.**
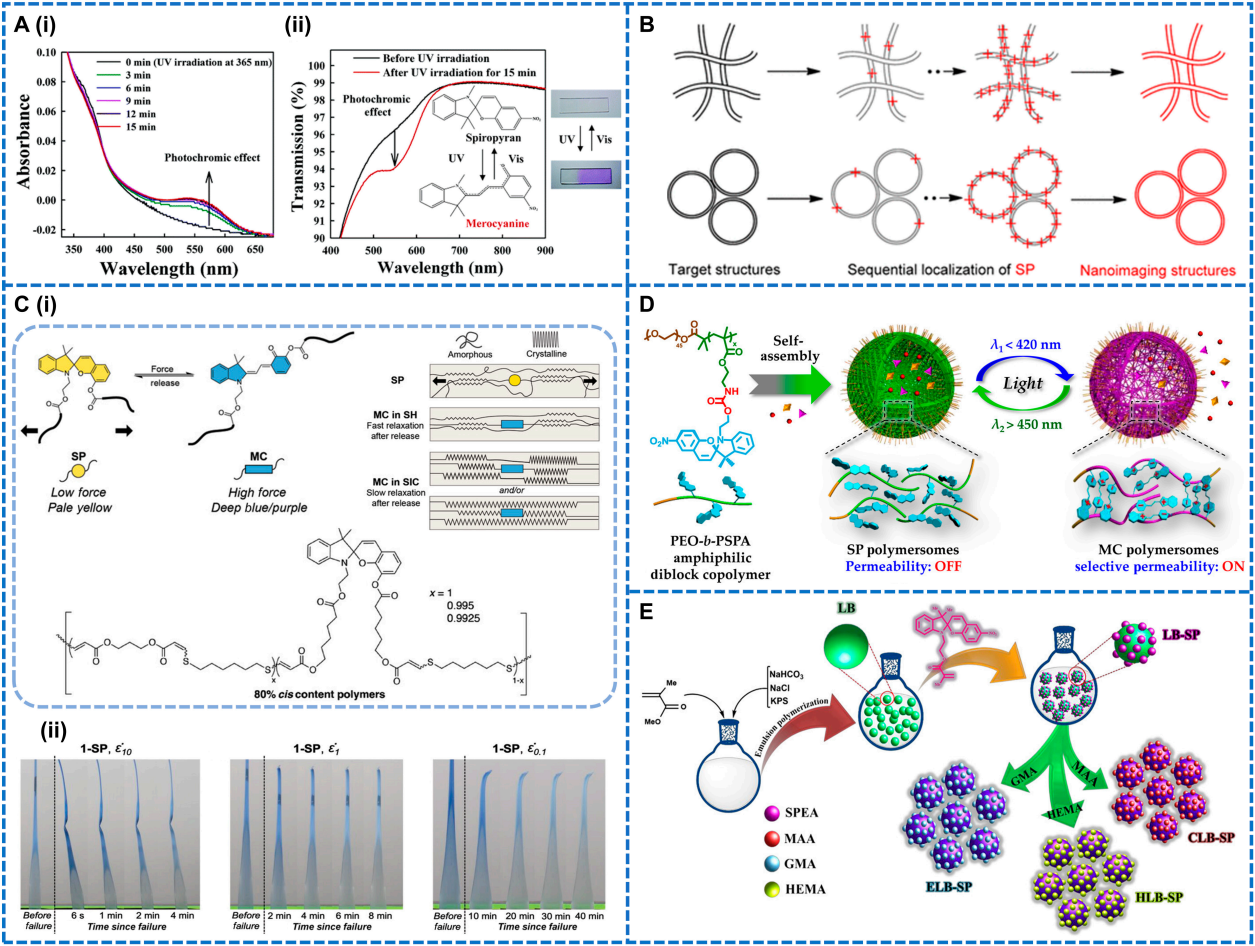
(A-i) Change in UV−vis spectra of SP-loaded (PS-b-P4VP/PS-b-PAA) multilayer films with increasing UV irradiation time at 365 nm. (A-ii) Light transmission curve of (PS7K-b-P4VP/PS-b-PAA) multilayer films before and after UV irradiation. Reproduced with permission from [67]. Copyright 2006, American Chemical Society. (B) Schematic of optical nanoimaging for microphase structures of BC self-assembly. Reproduced with permission from [72]. Copyright 2015, American Chemical Society. (C-i) Chemical structure of the polymer. (C-ii) Mechanochromism in semicrystalline polymers. Reproduced with permission from [73]. Copyright 2023, John Wiley & Sons Inc. (D) Photochromic polymers with photoswitching properties. Reproduced with permission from [74]. Copyright 2015, American Chemical Society. (E) Preparation of the functional photochromic latex particles containing SP by emulsifier-free emulsion polymerization. Reproduced with permission from [21]. Copyright 2018, American Chemical Society.

When SP photoresponsive groups are present in the polymer molecule, the polymer becomes a photoresponsive polymer. SP polymer films are usually characterized by excellent photoresponsive properties, stability, and tunability, and the film preparation process is simple and inexpensive; therefore, SP polymer films with excellent photoresponsive properties have been reported many times in recent years. In 2006, researchers doped water-insoluble SP molecules into the polystyrene (PS) core of BC micelles (BCMs) in multilayer films to fabricate multifunctional nanoporous films with antireflective and photochromic properties [67]. This film was assembled layer by layer by using electrostatic and hydrogen bonding interactions between different BCMs, including polystyrene block poly(4-vinyl pyridine) (PS-b-P4VP) and anionic polystyrene block poly(acrylic acid) (PS-b-PAA). Upon exposure to UV light at a wavelength of 375 nm for 15 min, the SP-containing films showed a decrease in the intensity of the absorption peak at 367 nm and the appearance of a new absorption peak at 550 nm, which increased in intensity along with time. This suggested that the discoloration of SP under UV irradiation was a result of C–O bond cleavage and *cis*-*trans* isomerization, which led to the formation of a parthenocyanine. The photoresponsive polymer could be restored by visible light irradiation (Fig. 5A-i and ii).

To date, polymer films have been investigated for use in a broad range of cellular fields [68], but the reversible control of surface property changes [69], as well as the cell adhesion and separation without the release of any molecules, have been hardly achieved [70]. A light-responsive polymer soft interface was produced that could reversibly control cell adhesion and separation in time and space [71]. The hydrophobic block of polymethyl methacrylate (PMMA) and the hydrophilic block of polyethylene glycol (PEG) were combined to form an amphiphilic diblock copolymer named P(SpMA-co-MMA)-b-PEG. SP molecules were incorporated into the hydrophobic block of this copolymer. The photoresponsive films of the SP-containing copolymers turned purple upon UV irradiation and reverted to transparency upon visible light irradiation, showing reversible photoisomerization between SP and MC. Yan et al. [72] were able to achieve SP photochromism and fluorescence switching by integrating SP as a coloring agent into hydrophobic BCs to prepare films (Fig. 5B). The colorless films containing SP (SPTS-PSt-b-PEO) turned purple at 365-nm UV irradiation in 5 to 10 s and fluoresced strongly in the deep red region at 670 to 690 nm. The films exhibited repeated reversible photochromism and fluorescence of SP, which was achieved by sequential UV and visible light irradiation. As a result, when used in hydrophobic polymer media, SPTS-PST-b-PEO with fast response and good reversibility was a suitable colorant for super-resolution optical imaging. A semicrystalline, recyclable, and mechanically discolored polymer was prepared by doping SP into elastomers of mercaptoalkyne derivatives (Fig. 5C-i) [73]. The modulus, strain hardening and strain-induced crystallization of the polymer depended on the strain rate (*ε*). When stress was applied to the elastomer, SP was activated to MC, and mechanical color change occurred. After the stress was removed, the polymer remained in the activated state for 1 h before returning to its colorless SP, and the time to return to the colorless state was dependent on *ε*. The degree of strain-induced crystallization increased with decreasing strain rate, while a low *ε* produced a greater color change, and the color lasted from 10 min to a few hours (Fig. 5C-ii). Subsequently, Wang et al. [74] reported photochromic polymer vesicles with photoswitching and bidirectional membrane permeability based on a newly designed poly(ethylene oxide)-b-PSPA dimer copolymer (PEO-b-PSPA, PEO-b-PSPO, and PEO-b-PSPMA) (Fig. 5D). The SP portion of the self-assembled polymer bilayer underwent a photoinduced reversible transformation between the hydrophobic spirulina (SP, *λ*_2_ > 450-nm irradiation) and amphiphilic anthocyanin (MC, *λ*_1_ < 420-nm irradiation) states. The self-assembled polymer with this amphiphilic structure was present in the form of micelles. Under UV irradiation, the hydrophobic spirulina molecules were converted into hydrophilic oleanolide molecules, which disintegrated the micelles. When exposed to visible light with a wavelength of 620 nm, the cells recovered and could be cycled 5 times. Accompanied by a reversible light-triggered transition from SP to MC polymers, the membrane changed from a nonpermeable state to the state of selective permeation of charged and amphiphilic small molecules below a critical molar mass.

SP polymer films exhibit excellent photochromic properties, including high contrast, fast response, and good reversibility. However, their stability and biocompatibility need improvement. In contrast, polymer NPs are of interest due to their stable structure and excellent physicochemical properties. NPs with specific functionalities can be precisely synthesized by tuning the polymer length, surfactant, and solvent.

#### Polymer NPs

Although SP polymer films have good photoresponse, their fatigue resistance is poor, and long-term light exposure will reduce the performance of the films and limit their application range. Therefore, researchers have discovered SP polymer NPs with excellent fatigue resistance [75], in which SP is chemically doped into hydrophobic or low-polarity polymers to improve their photochromic efficiency [76], such as in terms of light stability and photoreversibility. The photochromic polymers can also be modified by functionalized monomers, and the photochromic polymers can be chemically attached to different substrates to reduce the effects of environmental degradation. In recent years, photochromic polymeric NPs containing the SP fraction have been extensively investigated because of their photochromic effect and photoluminescent optical switching. In the beginning, there were a few studies on NPs capable of photoluminescent switching. In 2006, SP was incorporated into the hydrophobic cavity of polymeric NPs using NIPAM, styrene (St) as the main monomers and divinyl benzene as the cross-linking agent. Ethyl acrylate (EA) was used as the optically active moiety [77]. Polymeric NPs (NIPAM-St-EA) are formed by emulsion polymerization, and they were found to show a photochromic effect. The 68-nm NPs appeared blue upon UV irradiation and became colorless under visible light. The luminescence of NPs could be optically toggled by particular wavelengths of light.

Recently, the field of photochromic NPs has seen rapid development due to its rapid development. Color-changing dyes such as SP have been copolymerized with other monomers via emulsion or microemulsion polymerization to produce polymeric NPs that can be used in a variety of applications. Several polymeric NPs formed by different emulsion polymerization methods are described below. The researchers used a semicontinuous emulsion polymerization method to prepare SP photochromic nanoemulsions containing epoxy groups in 2015 [11]. It was found that with an increase of 1′-(2-acryloyloxyethyl)-3′,3′-dimethyl-6-nitrospiro-(2H-1-benzopyran-2,2′-indoline) (SPEA) monomer, the particle size in the range of 600 to 700 nm increased, while the absorption intensity decreased with the increase of particle size. The best photochromic performance was obtained with a concentration of SPEA at 3%, which made the NPs turn purple under UV irradiation. The photochromic properties were then investigated by impregnating cellulose paper with photochromic latex. After drying at 80 °C and then immersing in water, ethanol, and methanol of different polarities, a piece of photochromic paper was prepared. The paper showed a blue-purple color before and after UV irradiation when soaked in water, a purple color in ethanol, and a pink color in methanol. This phenomenon could be ascribed to the interplay between colored MC forms in diverse polar environments, leading to the absorption of light with varying wavelengths and consequent exhibition of distinct colors.

Later on, to study nontoxic latex particles without additives, Abdollahi and coworkers went on to develop solvent-free attachable polymeric anti-counterfeit inks based on photochromic latex particles containing SP for the first time in 2018 [21]. The primary focus of previous research on SP-based photochromic polymers was on the synthesis of polymeric NPs using techniques such as emulsion polymerization, fine emulsion polymerization, and emulsifier-free emulsion polymerization. Here, methyl methacrylate (MMA) and 1% SPEA were used to synthesize functionalized stimuli-responsive latex particles (LB-SP), the carboxylated photochromic latex particles (CLB-SP), hydroxylated photochromic latex particles (HLB-SP), and also epoxidized photochromic latex particles (ELB-SP) containing SP by semicontinuous soap-free emulsion polymerization (Fig. 5E). Incorporation of MC molecules onto the surface of SP-doped latex particles resulted in a decrease in particle size and an increase in surface tension. This change in particle size could be controlled and reversed by alternating exposure to UV and visible light. Upon UV irradiation, the initially colorless SP form was converted to the colored MC form, characterized by a broad absorption peak spanning the 400- to 700-nm range. The latex particles were found to exhibit good phototransformability after each cycle and became photofatigued after 15 cycles. In addition, the NPs showed red fluorescence after UV irradiation. However, the negative photochromism and reduced photoresponse of the latex particles were the drawbacks of this study.

Based on the above research, Alidaei-Sharif et al. [78] prepared latex NPs for dual photochromic and photoluminescent security inks (MMA-HEMA-SPOH), by chemically doping SP into copolymer latex NPs of MMA and hydroxyethyl methacrylate (HEMA). The SP molecule could be isomerized between the SP and MC forms due to its photochromic properties and emitted red fluorescence after absorbing UV irradiation. With a decreasing particle size, the polar photochromic and photoluminescent properties were enhanced. SP NPs were found to show long-term photoswitching and fatigue resistance and could be irradiated for 40 cycles in UV–visible light. Therefore, it could be used to prepare an anti-counterfeiting ink. However, the main shortcoming of this study was that the NPs swelled with increasing HEMA content, thus affecting the photochromic and luminescent properties. Negative photochromism and reduced photoresponse of latex particles were also the drawbacks of this study. To date, mitigating the negative effects of photochromism and enhancing photostability and reversibility have been major hurdles for photochromic polymers. Future investigations should prioritize strategies aimed at reducing negative photochromism while improving photoreversibility and photostability. To investigate how to reduce negative photochromism, researchers used semicontinuous microemulsion polymerization to develop flexible anti-counterfeit inks by chemically doping SP into a copolymer emulsion of NPs based on MMA and butyl acrylate (BA) [79]. The resulting PBAMMA-SP2 (67% MMA and 33% BA) was moderately flexible. The polymer chains were polarized and exhibited fast photoresponsiveness to UV and visible light. PMMA-SP (PMMA film) also showed high-intensity photochromic, bright red fluorescence, light switching, and light fatigue resistance without negative photochromism. The photochromic anti-counterfeiting ink prepared from PMMA-SP samples was mounted on different stamps coated on security documents for printing markings. They could also be used to collect fingerprints on passports as security markings. This could be done by spraying the ink on cellulose paper, UV irradiating for 1 min with different masks, then removing the masks to view the colored photopatterns. The photopatterns were fully reversible under multiple cycles of alternating UV and visible light irradiation.

The research on SP polymers is now very extensive but how to better apply their photochromic and photoluminescent properties remains as a question for consideration. Some applications of SP polymers will also be reviewed in subsequent sections of this review.

### Applications of SP photochromic polymers

SP photochromic polymers are novel materials that have found a series of applications in reversible and rewritable data storage or printing [80], anti-counterfeiting systems [81], drug delivery [82], high-resolution bioimaging, chemical sensors [83], switchable fluorescent polymer particles, cell labeling, and ophthalmic lenses [84]. SP photochromic polymers are biocompatible and renewable, making them environmentally friendly materials with a promising future. In addition, SP photochromic polymers can also be used as novel optical materials for the manufacture of optical devices and optical braking system [85]. In the following, the applications of SP photochromic polymers will be reviewed in 3 major areas:

#### Anti-counterfeiting ink applications

**Fig. 6.**
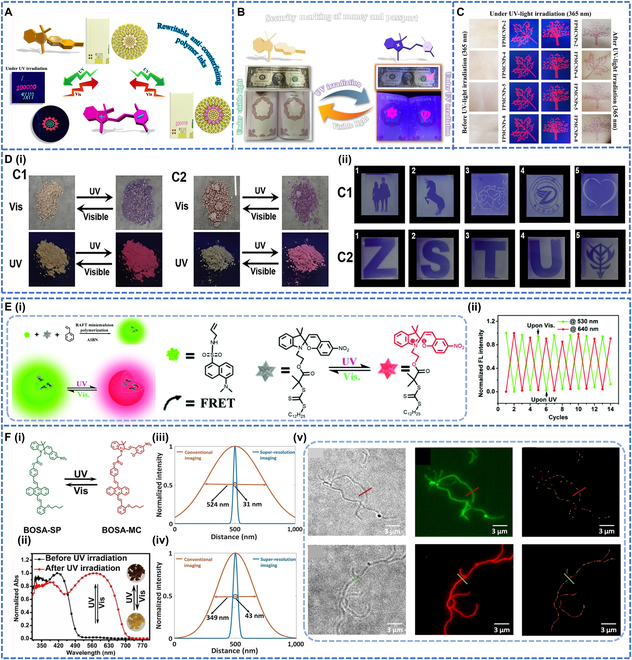
(A) Application of the functionalized stimuli-responsive latex particles (CLB-SP) as an anti-counterfeiting and rewritable smart ink for secured marking in security documents. Reproduced with permission from [21]. Copyright 2018, American Chemical Society. (B) Security printing of photoluminescent and photochromic anti-counterfeiting inks on confidential documents such as banknotes and passports. Reproduced with permission from [78]. Copyright 2023, Elsevier Ltd. (C) Printed optical security tags by using photoluminescent and photochromic NPs containing SP (FPMCNPs-SPOH); the photography was carried out before, during, and after UV light illumination (365 nm). Reproduced with permission from [88]. Copyright 2023, American Chemical Society. (D-i) Color and fluorescent images of C1 and C2 powder. (D-ii) Schematic illustration of write-erase cycles carried out on films containing C1 and C2 Reproduced with permission from [87]. Copyright 2021, Elsevier Ltd. (E-i) Schematic illustration of photoswitching behavior of PFPNs under UV and visible light irradiation. (E-ii) Photoinduced switching cycles of PFPNs (NP-N3) under alternative illumination of UV for 3 min and visible light for 5 min (*λ*_ex_ = 410 nm, 25 °C). Reproduced with permission from [91]. Copyright 2017, Royal Society of Chemistry. (F-i) Reversible structure isomerization of BOSA-SP and BOSA-MC. (F-ii) UV/Vis absorption spectra of BOSA-SP powders before and after UV irradiation. (F-iii) Green and (F-iv) red conventional fluorescence and super-resolution imaging of the cross-sectional profiles of the PSt-b-PEO cylindrical micelles at the dashed lines of the microscopy images. (F-v) Polymer super-resolution imaging applications. Reproduced with permission from [92]. Copyright 2022, John Wiley & Sons Inc.

In terms of anti-counterfeiting systems, SP photochromic polymers can increase the security of the system and provide more reliable protection against counterfeiting due to their unique and wide range of color stimulating properties. SP polymers can be used as security inks for erasable patterns, optical data storage, and security markers [86]. Among different types of applications, anti-counterfeiting is the most widespread type. Abdollahi et al. [21] reported SP polymer-based anti-counterfeiting and erasable inks based on photochromic latex particles. SP polymer latex particles were used as a security ink for writing on cellulose paper. The recorded words exhibited reversible red fluorescence and color changes when exposed to cyclic UV and visible light radiation, which was manifested a photoinduced change from SP to MC. As the ink was solvent-free, its large particle size allowed it to be stable on the paper surface. The ink could be sprayed onto cellulose paper to prepare a piece of photochromic paper, and the resulting stimulus-responsive paper exhibited outstanding reversible photopatterning capabilities under diverse masks and UV irradiation. Latex particles could hence be used for anti-counterfeiting and security markings (Fig. 6A).

Subsequently, Alidaei-Sharif et al. [78] used 80% PMMA and 20% HEMA as security inks to make stamps of balloons, butterflies, and leaves, which changed color and emitted red fluorescence before and after UV irradiation. The sample was loaded into a pen for writing on cellulose paper to obtain writings with reversible color changes before and after UV–visible light irradiation. The ink could also be loaded in an automatic stamp to print different security labels with marked reversible photochromic and fluorescence changes under UV irradiation (Fig. 6B).

In addition to photochromism and photoluminescence, SP polymers have also been investigated for mechanical color changes in anti-counterfeit inks. The researchers synthesized 2 new SP-based photochromic molecules with different silane moieties, which could achieve both reversible photochromism and mechanical color changes [87]. The color of compounds C1-C2 hanged from light yellow to purple under UV irradiation, and the original color was restored by visible light irradiation, demonstrating the reversible isomerization between SP and MC (Fig. 6D-i). This photochromic molecule was dispersed in polyvinylpyrrolidone and spin-coated onto a film. An image was printed on the film using a mask. The image was found to be of high resolution and remained colorfast for at least 2 d and fatigue-free for at least 40 cycles. It was used as an anti-counterfeiting ink to print patterns on paper using a photochromic solution (Fig. 6D-ii).

Previous studies have also been conducted on single photochromic or photoluminescent NPs, and bi- or multicolor photoluminescent color-changing NPs represent a class of novel anti-counterfeiting materials that has been extensively studied in security anti-counterfeiting inks. The coblending of SP and coumarin by emulsion polymerization was first reported, resulting in the synthesis of functionalized polymeric NPs. These NPs exhibited various functional groups, including tertiary amines and amides [88]. The synthesized NPs exhibited time-dependent (dynamic) fluorescence emission in 2 or multiple colors triggered by a single wavelength of light. These NPs were utilized as photochromic inks for counterfeit deterrence, enabling the printing of secure labels on cellulose paper using a stamp printer. The labels were invisible in visible light, and the color changed to purple with red fluorescence when illuminated by UV light at 365 nm. This static–dynamic dual-color photoluminescent ink possessed the advantages of dual-color fluorescence emission, dynamic photochromic, invisibility under environmental conditions, strong printability, multilevel security, high resolution and luminance, etc. This work thus opened up new paths for future intelligent anti-counterfeiting technology (Fig. 6C).

#### Bioimaging and super-resolution imaging

The closed form of SP changes to the open form MC upon UV excitation, and this process is usually accompanied by red fluorescence [89]. It was found that SP photoswitches modified with different substituents would exhibit dual fluorescence color-changing properties in the aggregated state, with the most important applications in the fields of biological imaging and super-resolution imaging [90]. Zhu et al. [77] used NPs for imaging by placing them in selected wells of a 96-well microtiter plate to produce speckle maps. Under UV light irradiation, a red-emitting light pattern was shown. The pattern disappeared completely after 30 min of standing under visible room light. The photoerased pattern reappeared when the object was exposed to UV light. The possibility of optical switching in vivo was observed by delivering the NPs into living cells. Using a liposome as a carrier system, optical control of the NPs was achieved in HEK-293 cells, allowing for precise delivery. When excited with a 365-nm Ar laser, short UV (293 nm) pulses turned on the fluorescence of the polymeric NPs in live HEK-488 cells. As the exposure time increased, the red fluorescence of the particles became weaker. The fluorescence turn-on process was rapid, in which the red fluorescence could be readily triggered within 10 s of UV light exposure without apparent cell damage.

Later, Chen et al. [91] used the dan-sulfonamide derivative as a fluorescence donor, SPTTC (Sulfur-Containing Primary Transfer Chain Transfer Agent) as a chain transfer agent and a photochromic acceptor in a single-pot RAFT-mediated microemulsion polymerization to prepare a new type of 2-color photoswitchable fluorescent polymer nanoparticles (PFPNs) (Fig. 6E-i). The PFPNs showed high 2-color contrast, fast responsiveness, and photo reversibility. The 2-color cycle could be repeated a minimum of 7 times (Fig. 6E-ii). PPNPS were therefore used for photoerasable fluorescent patterning and intracellular 2-color fluorescent imaging. Upon UV irradiation, the NPs showed red fluorescence due to isomerization; while upon visible light irradiation, no red fluorescence was observed due to the nonfluorescent SP state.

Ordinary SP polymers exhibit only red fluorescence and were not yet capable of showing multicolor fluorescence or color-changing transitions. To achieve super-resolution imaging with different fluorescence color transitions, Yang et al. [92] prepared the novel fluorescent photoswitches, BOSA-SP, which could achieve green, yellow, and red fluorescence under pump light excitation and triggered light-induced isomerization (Fig. 6F-i and ii). The substantial free space within the solid state of the SP moiety facilitated reversible photoisomerization, enabling its application in 2-color super-resolution imaging. In this study, BOSA-SP was incorporated into a diblock copolymer, specifically polystyrene-block-poly(ethylene oxide) (PSt-b-PEO), resulting in the formation of micellar assemblies. Different filters were selected for imaging to obtain micelles with green and red fluorescence (Fig. 6F-v). Super-resolution image processing was achieved to improve the image quality down to 31 nm for green fluorescence and down to 43 nm for red fluorescence (Fig. 6F-iii and iv), allowing simultaneous collection of multiple spectra in both green and red fluorescence channels.

#### Other applications

**Fig. 7.**
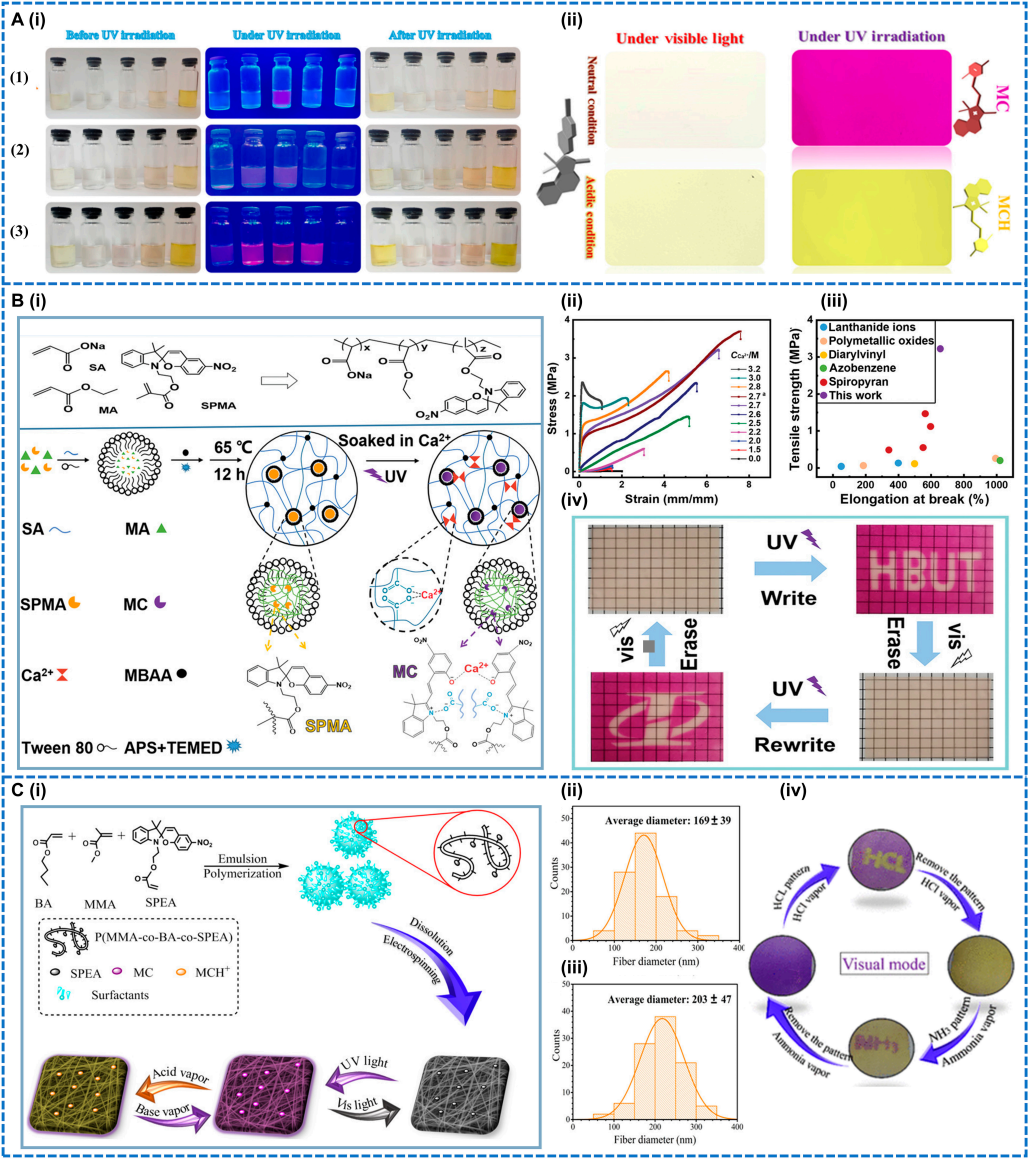
(A-i) Photochromism before, under, and after UV irradiation (365 nm) for the (1) PMMA, (2) PMMA-co-PHEMA, and (3) PMMA-co-PDMAEMA NPs. The pH of all the samples is 1, 3, 7, 10, and 14, from left to right, respectively. (A-ii) The coloration of the photochromic cellulosic papers under UV (365 nm) and visible light irradiation in neutral and acidic conditions. Reproduced with permission from [93]. Copyright 2022, American Chemical Society. (B-i) Synthetic scheme of ion-hybrid cross-link photochromic hydrogels. (B-ii and iii) tensile stress–strain curves of P(SA-co-MA-co-SPMA)/Ca^2+^ photochromic hydrogels soaked in solutions at different Ca^2+^ concentrations. (B-iv) The process of rewritable imaging on the P(SA-co-MA-co-SPMA)/Ca^2+^2.7 M hydrogel by using UV light. Reproduced with permission from [94]. Copyright 2021, John Wiley & Sons Inc. (C-i) Schematic representation for preparation of MBSP NPs and the corresponding stimuli-responsive nanofibers with color changes. (C-ii and iii) Diameter distribution of 2 types of nanofibers. (C-iv) Images of the MBSP@NF sheet before and after exposure to HCl and ammonia vapors using “HCL” and “NH_3_” patterns in their visual mode. Reproduced with permission from [95]. Copyright 2020, American Chemical Society.

In recent years, with the widespread reports of photochromic polymers, researchers used copolymerized SP functional monomers with PMMA to form photochromic polymeric NPs, PMMA-co-PHEMA and PMMA-co-PDMAEM [93], average size of which was below 100 nm. Photochromic quenching occurred when the NPs were placed at pH 1 or 14, due to the generation of highly protonated MC and highly deprotonated MC in the presence of strong acids and bases, which exhibited photochromic phenomena when placed at pH 3, 7, and 10. The highly sensitive pH responsiveness exhibited by the NPs could be used as a chemical sensor (Fig. 7A-i). A piece of cellulose paper impregnated with PMMA-co-PDMAEMA was used for the detection of acid vapor, and it was found to change color completely under UV irradiation, demonstrating its application as a chemical sensor for acid vapor (Fig. 7A-ii).

Ion-hybrid cross-linked hydrogels with photochromic properties have been synthesized by incorporating acrylate derivatives of SP functional groups (SPMA) into poly using a micelle copolymer technique (Fig. 7B-i) [94]. The mechanical properties reached a maximum at a Ca^2+^ concentration of 2.7 M with a tensile strength of 3.22 MPa, an elastic modulus of 8.6 MPa, and a tensile work of 12.8 MJ m^−3^ (Fig. 7B-ii and iii). Upon exposure to 365-nm UV light, the hydrogel underwent a spectral transformation, revealing a distinct absorption peak at 558 nm. After 30 s of UV irradiation, complete SP-MC isomerization was achieved, and the 558-nm peak disappeared after approximately 6 h of subsequent visible light irradiation. The hydrogels exhibited extraordinary properties of reversible light conversion and exceptional durability against light-induced fatigue, making them highly promising for a wide array of applications such as optical devices, reusable optical data storage, artificial intelligence systems, and adaptable wearable devices (Fig. 7B-iv). In addition, Rad et al. [95] prepared photoresponsive polymers by copolymerization of SPEA with MMA and BA using emulsion polymerization and prepared nanofibers and films (MSP@NF and MBSP@NF) using electrostatic spinning and drop casting techniques (Fig. 7C-i). The *T*_g_ of the photoresponsive poly(MMA-co-SPEA) (MSP) copolymers was slightly elevated (~10 °C) due to the chemical doping of SPEA into the copolymer chains. The morphologies of nanofibers in MSP@NF and MBSP@NF were smooth and homogeneous, with 85% and 89% porosity and average diameters of 169 and 203 nm, respectively (Fig. 7C-ii and iii). MBSP@NF with a high photoswitching rate was selected for optical patterning, and the polymer color was changed from violet to whitish-yellow under 365-nm UV irradiation in the presence of HCl vapor, which could be repeated for 10 cycles (Fig. 7C-iv). The photoswitchable write-erase pattern on the surface of the MBSP@NF confirmed its capability for optical patterning and real-time data storage.

In brief, the applications of SP polymers are not limited to the ones we have mentioned here, and the development of more attractive applications is still a major focus of researchers, hopefully for medical equipment and transducers in the future.

## Diarylethene

### Overview

DAE and its derivatives undergo not only *cis*-*trans* isomerization but also reversible photocyclization under UV irradiation, switching between the colorless open and colored closed ring isomers. Both isomers exhibit strong fatigue resistance and are thermally stable. The switching of both isomers is reversible and can be repeated hundreds or thousands of times [22]. In 1988, Irie and coworkers [96] synthesized heterocyclic substituted DAEs with good photochromism, thermal stability, and fatigue resistance and fast response, through which we can know that the photochromism mechanism is based on a photocyclization reaction. Under photoexcitation by UV light, the compound rotates to close the ring and produce a colored closed-ring structure, which can undergo the opposite ring-opening reaction under visible light irradiation.

With their excellent photoreactivity, thermal stability, and fatigue resistance, diarylethylenes can be used in optical storage devices [97], fluorescence sensing [98], bioimaging [99], construction of logic gates, and so on. There are various types of aromatic heterocycles of diarylethylenes, and diarylethylenes containing 2 thiophene-derived groups are of the most interest because they are suitable for as switching units. There are some reviews on photochromic DAE polymers, and this review will mainly focus on several common classes of DAE photochromic polymers and their applications.

### Classification of DAE polymers

#### Nonheterocyclic substituted DAE

**Fig. 8.**
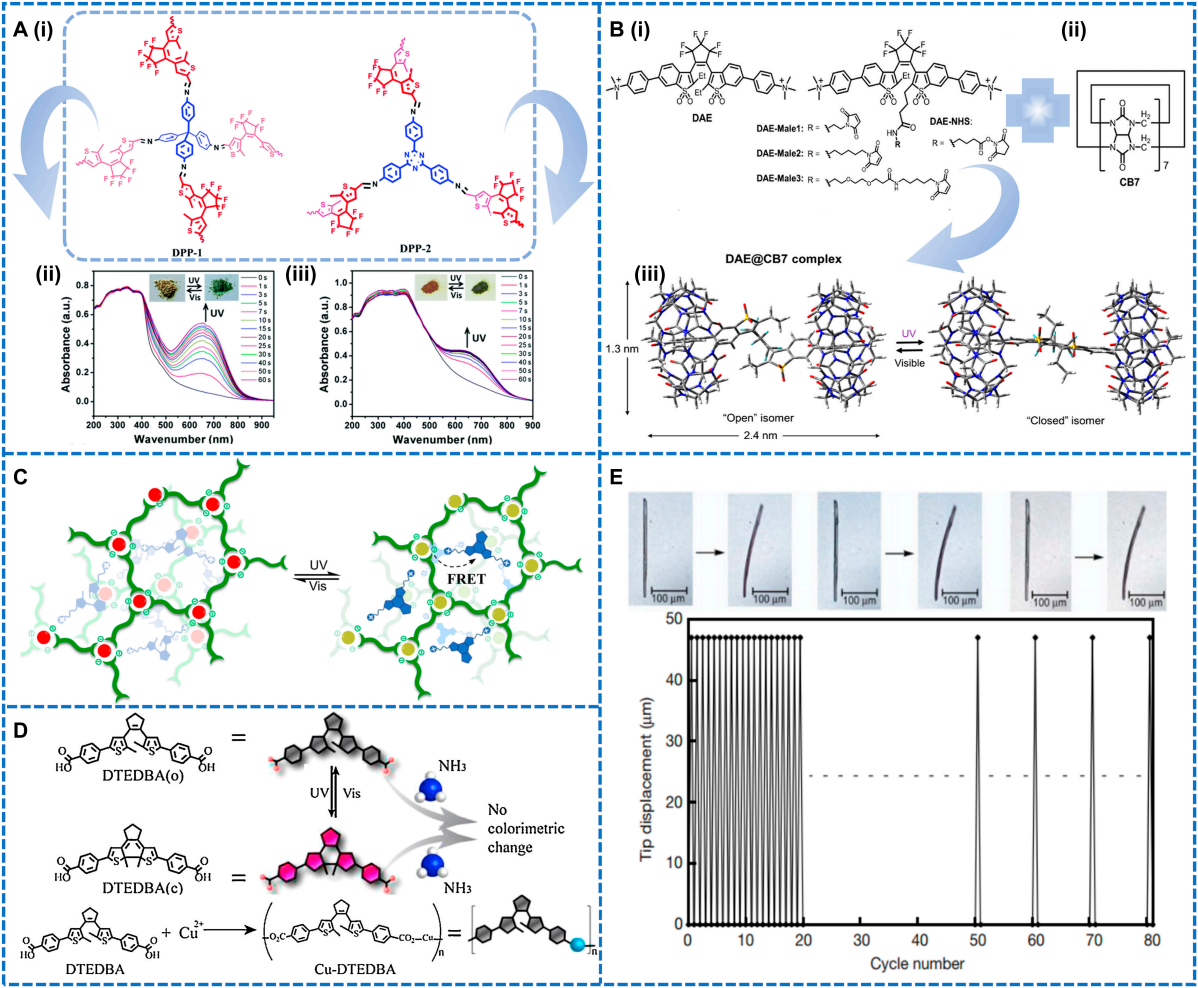
(A-i) Illustration of the synthesis of DPP-1 and DPP-2 by the Schiff-base polycondensation reaction. Time-dependent UV–vis–NIR absorption spectral changes of (A-ii) DPP-1 and (A-iii) DPP-2 upon irradiation with 365-nm light. Reproduced with permission from [100]. Copyright 2019, Royal Society of Chemistry. (B-i) Structures of photochromic DAEs as guest molecules (DAE, DAE-Male1, DAE-Male2, DAE-Male3, and DAE-NHS). (B-ii) Structure of a cucurbit [7] uril host molecule (CB7), (B-iii) DFT-optimized geometry and the photoswitching reaction of the DAE@CB7 complex. Reproduced with permission from [101]. Copyright 2022, American Chemical Society. (C) The construction of the photochromic SCP, and the chemical structures of corresponding components. FRET, fluorescence resonance energy transfer. Reproduced with permission from [102]. Copyright 2021, Springer Nature. (D) Photoisomerization of DTEDBA and Cu-DTEDB. Reproduced with permission from [105]. Copyright 2016, John Wiley & Sons Inc. (E) Reversible bending of a crystalline rod. Reproduced with permission from [103]. Copyright 2007, Springer Nature.

Nonheterocyclic substituted diarylethylene refers to compounds in the diarylethylene molecule where no heterocyclic groups, such as thiophene and pyridine, have been introduced on the aromatic hydrocarbon moiety. These compounds exhibit specific optoelectronic properties and reactivity by linking the 2 aromatic hydrocarbon groups through a vinyl group. Thermally durable photochromic conjugated polymers, namely DPP-1 and DPP-2, were synthesized by polymerizing dopamine with DEA-based dialdehydes as photochromic moieties (Fig. 8A-i) [100]. Upon exposure to 365-nm UV light for 50 s, DPP-1 exhibited a color transition from a pale yellow shade to a vibrant grass green, while DPP-2 changed from yellow to an earthy olive green. The color recovery of both compounds was achieved under visible light (Fig. 8A-ii and iii). Afterward, 2 polymer films were prepared using PMMA and the color of DPP-1/PMMA film changed from pale yellow to grass green within 5 s under UV irradiation, while DPP-2/PMMA film changed from yellow to olive green within 50 s. The color of the UV–visible irradiated film changed reversibly. This leads to the conclusion that the introduction of photochromic DAE into the conjugated network allowed the preparation of fast photoresponsive photochromic polymers.

Future applications of DAE-based polymers to optics are yet to be investigated. In a recent study, sulfone DAEs with simple structures and high fluorescence quantum yields for fluorescence turn-on properties were used for photoswitching. Jin et al. [101] designed a supramolecular system (DAE@CB7) with a photoswitchable fluorescent sulfone DAE as a guest and a cucurbit urate (CB7) as a host. In this system, photoswitchable DAE molecules were enclosed by CB7 to form a host–guest pair, in which the CB7 moiety could protect the DAE from the environment and improved its fluorescence intensity and ability to withstand fatigue in pure water. Under intermittent UV and visible light exposure, the process of photoisomerization could be iterated up to 2,560 times in an aqueous solution before partial fading transpired, whereas free DAE could only be switched on and off 80 times. DAE@CB7 was prepared by doping with reactive groups (maleimide and *N*-hydroxysuccinimide [NHS] esters) and it could be used for the specific labeling of intracellular proteins as well as reversible on/off switching of the probe in a cellular environment under 355-nm/485-nm light irradiation (Fig. 8B-i to iii). Li et al. [102] reported the hierarchical self-assembly of lanthanide ions, bilayers, and DAE units driven by metal–ligand and ion interactions to construct photoresponsive supramolecular coordination polyelectrolytes (SCPs) (Fig. 8C). The closed/open ring isomerization of the DAE unit led to the photoreversible luminescence conversion of the SCP. The colorless solution of the polyelectrolyte changed to dark blue upon UV irradiation and the color returned to colorless upon visible light irradiation, proving that the photoisomerization was reversible. After 4 consecutive cycles of alternating UV and visible light irradiation, there was no marked decrease in luminescence intensity. Thus, this polyelectrolyte exhibited excellent fatigue resistance and could be used for anti-counterfeiting purposes. The unique photochromic reactions of DAE molecules, especially in their single-crystalline phase, have not been extensively investigated. In this context, the authors focused on molecules like 1,2-bis(2-ethyl-5-phenyl-3-thienyl) and so on. These molecules undergo thermally irreversible and fatigue-resistant photochromic reactions in both solution and single-crystalline phases. This reaction could lead to single-crystal structure deformations upon alternating irradiation with UV and visible light (Fig. 8E) [103]. This innovation paves the way for the development of advanced materials capable of controlled deformations upon light irradiation.

#### Heterocyclic-substituted DAE

Thiophene ring-substituted DAEs are the most commonly studied heterocyclic DAEs due to their unique optoelectronic properties and potential applications in organic optoelectronic devices and photochromic materials. Dithienylethylenes (DTEs), developed by Irie and coworkers, represent one of the most widely reported class of photochromic compounds [104].

The new photochromic aggregation-induced luminescence (AIE) active polymer (DTE-TPE-AIE) were synthesized containing DTE and tetraphenylethylene (TPE) fractions via a condensation reaction [104]. The diphenyl ethylene chromophores in the polymers could undergo photoisomerization between their open and closed forms when irradiated with UV and visible light. The photochromic properties of the polymers were investigated in solution, while the photoswitching properties were investigated in solid films. Upon UV irradiation, the tetrahydrofuran solution containing the polymers turned blue and a new absorption band (622 nm) appeared, indicating that the DTE chromophores in the polymers formed closed-loop isomers. Under visible light irradiation, the blue tetrahydrofuran solution reverted to colorless, indicating that the compound reverted to the open-loop isomer. The photochromic properties of the polymers in the films were similar to those in solution.

Li et al. [105] doped diphenyl ethylene into an infinite coordination polymer, Cu-DTEDBA, to obtain a photochromic polymer. The light green Cu-DTEDBA powder turned dark blue within 5 min of UV irradiation and recovered within 30 min of visible light irradiation, and this behavior was attributed to the reversible photoisomerization of diphenyl ethylene. Synergistic effects between the DTE and ICPs structures allowed their use as chemical sensors to detect color changes. Upon exposure to gaseous ammonia, the open form of the polymer exhibited a rapid colorimetric change (within 3 s) from light green to dark cyan, and the closed form exhibited a change from dark blue to purple. Thus, both isomers could be used as gas probes (Fig. 8D).

### Applications of DAE polymers

#### Anti-counterfeiting applications

DAE-based polymers have shown great potential in the field of advanced information encryption and anti-counterfeiting of their information storage [106]. A light-emitting switch SCP with fast response, DAE good fatigue resistance, and thermally irreversible properties was designed. To further explore its application in smart security, SCP was used as a security ink to print high-resolution quick response codes, which were invisible in daylight and visible in bright red when illuminated by a 254-nm UV lamp and could be quickly scanned by a smartphone to retrieve information, enabling visible/invisible conversion of light-reversible multi-information patterns.

**Fig. 9.**
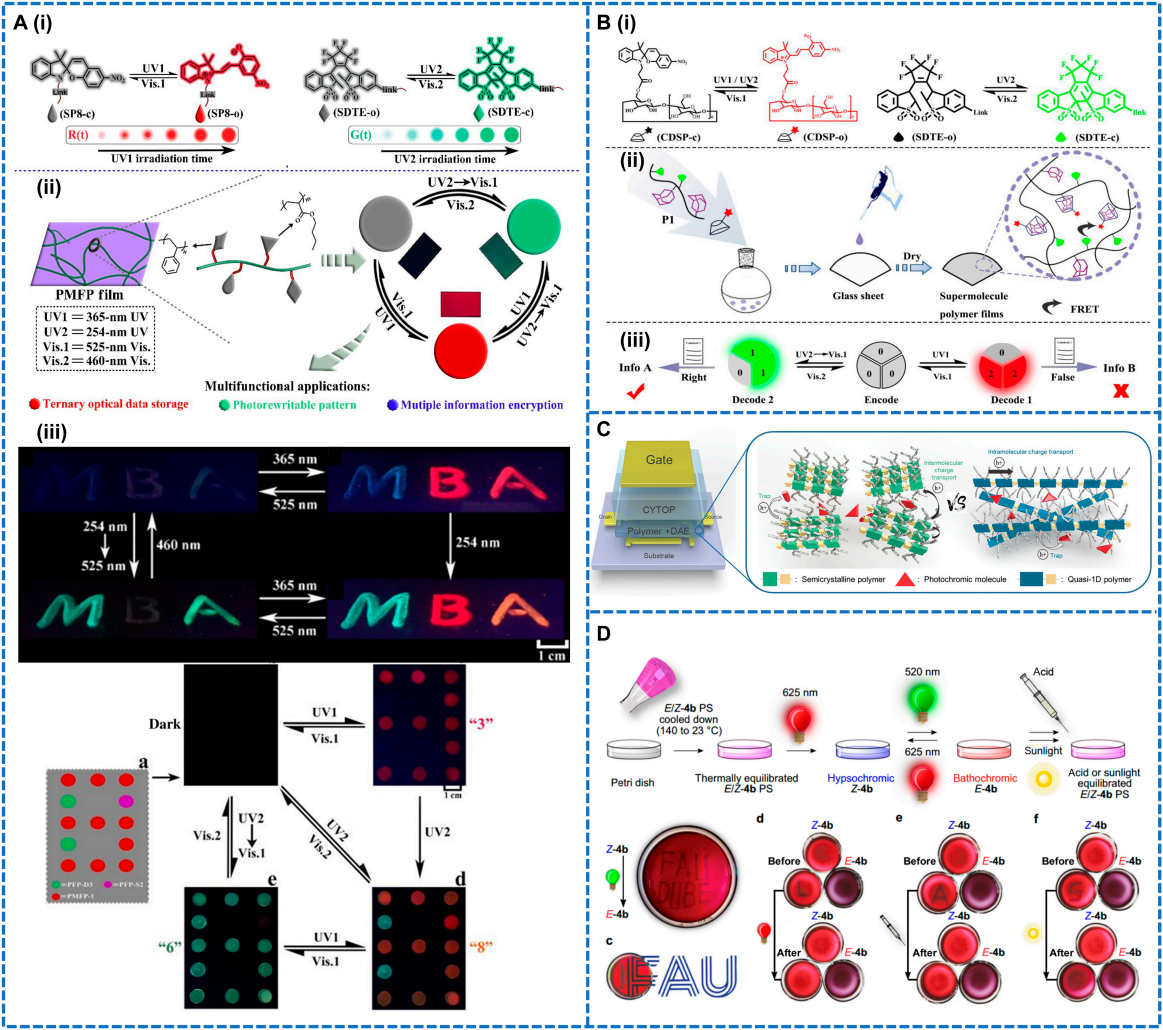
(A-i) Schematic illustrations of the design strategy from 2 photochromic fluorescent monomers to a photoswitchable multistate fluorescent polymer. (A-ii) The photoswitchable multistate fluorescent polymer can reversibly switch between multiple emission states (nonemission, red and green). (A-iii) Polymeric solid films for multi-information encryption and advanced anti-counterfeiting. Reproduced with permission from [109]. Copyright 2021, Elsevier Ltd. (B) Schematic diagram of PMFSPs: (B-i) photochromism of CDSP and SDTE, (B-ii) preparation of PMFSP films, and (B-iii) information encryption applications of PMFSPs. Reproduced with permission from [110]. Copyright 2023, American Chemical Society. (C) Device structure of optical switchable transistors. Reproduced with permission from [111]. Copyright 2023, John Wiley & Sons Inc. (D) Incooperation of diaryl-HI 4b into a PS polymer and resulting photochromic behavior of the transparent material. Reproduced with permission from [112]. Copyright 2023, Springer Nature.

With the development of technology, monochromatic fluorescence encryption [107] and anti-counterfeiting [108] can no longer meet the needs of people. To achieve encryption of different color fluorescence [105], Jiang et al. [109] prepared photoswitched polymorphic fluorescent polymers (PMFPs) by free radical copolymerization of BA and St with photochromic monomers, namely sulfonyl diaryl acrylate 4-hydroxy butyl ester (SDTE) and SP-linked methacrylate (SP8) (Fig. 9A-i and ii). Due to controlled fluorescence resonance energy transfer from the excited diaryl group to the SP fraction, the emission of the polymer in the film could be reversibly switched between nonemitting, red, and green fluorescent states. Based on their high brightness, high contrast, and fast optical response, the PMFPs could be used for multi-information encryption and advanced anti-counterfeiting applications. As shown in Fig. 9A-iii, PFP-S2, PFP-D3, or PMFP-1 solid films made of fluorescent polymers were coated on a circular glass surface. When there was no UV irradiation, the spots were nonemitting and constituted the encrypted state. When irradiated with 365-nm UV light, PFP-S2 and PMFP-1 showed red emission and the red number “3”. When irradiated with 254-nm UV light, the number changed to “8”, then with 525-nm visible light, the red emission turned off and the green number “6” appeared. The initial encrypted state could be restored by irradiation with 460-nm visible light. The polymer was then used as a security ink to write numbers on glass, with different excitation wavelengths producing fluorescence in different letters, such as red and green. The polymer could be used in the future to explore further applications for sophisticated anti-counterfeiting.

Subsequently, the use of supramolecular polymers and fluorescent photochromism to construct photoswitchable polymorphic fluorescent supramolecular polymers (PMFSPs) has been investigated. Tang et al. [110] prepared photoconvertible PMFSP using adamantane-containing functional monomer and photochromic fluorescent DTE monomer (SDTE) with structural monomers such as BA and MMA via radical copolymerization (Fig. 9B-i to iii). Under different light stimuli, the PMFSP exhibited marked triplet fluorescence jumps in the colorless, green, and red states. The PMFSP films showed little fatigue effect after more than 10 times of alternating UV–visible irradiations. Due to the high contrast, fast photoresponsiveness, and excellent light reversibility, PMFSPs could be used for advanced anti-counterfeiting and multilevel information encryption applications. Researchers have successfully fabricated mixed thin films and applied them to optically switchable field-effect transistors by DAE with quasi-1-dimensional semiconducting polymers (Fig. 9C) [112]. This innovative approach allowed DAE not only to capture intermolecular carrier transfer but also to complement intramolecular transfer processes. The application of DAE polymers demonstrated their innovative potential in the field of optoelectronics and provided a powerful solution for the development of high-performance and multifunctional optoelectronic devices. Recently, Sacherer et al. [112] delved into the realm of DAE, showcasing their potential as visible light-, pH-, and heat-responsive 4-state switches (Fig. 9D). Their research highlighted the application of these compounds in photochromic transparent polymer, including their ability to undergo structural transformations upon specific stimuli.

#### Logic gate applications

Photochromic polymers have been intensively investigated for integration into logic gates [113]. It is well known that among the photoswitchable molecules [114], DTE and DAE photochromic units are among the most promising candidates for a system that switches by light irradiation or by redox stimulation between 2 stable isomers [115]. Andréasson et al. [116] reported a photochromic ternary system consisting of DTE and 2 fungicides (FG) that could act as a single-molecule, multifunctional, and reconfigurable logic systems. Four isomers could be produced using different wavelengths, which were labeled as FGo-DTEo, FGc-DTEo, FGo-DTEc, and FGc-DTEc. For example, FGo-DTEo, whose thermal stability is the best, did not absorb more than 450 nm. When irradiated at 397 nm, the absorption was almost exclusively caused by FGo, isomerization was performed to a photostationary state composed predominantly of FGc-DTEo. The new band was caused by FGc, which emitted maximum fluorescence at 624 nm. Green light (460 < *λ* < 590 nm) reconverted FGc-DTEo into FGo-DTEo. Instead, if the FGo-DTEo solution was irradiated at 302 nm, with the majority of absorption by DTEo, the sample was isomerized to a photostationary state comprising predominantly FGo-DTEc. Similar interconversions between all 4 isomers could be achieved with light of different wavelengths. It was shown that these 4 isomers were photostable and thermally stable. Multiple conversions could be achieved to make logic gates.

**Fig. 10.**
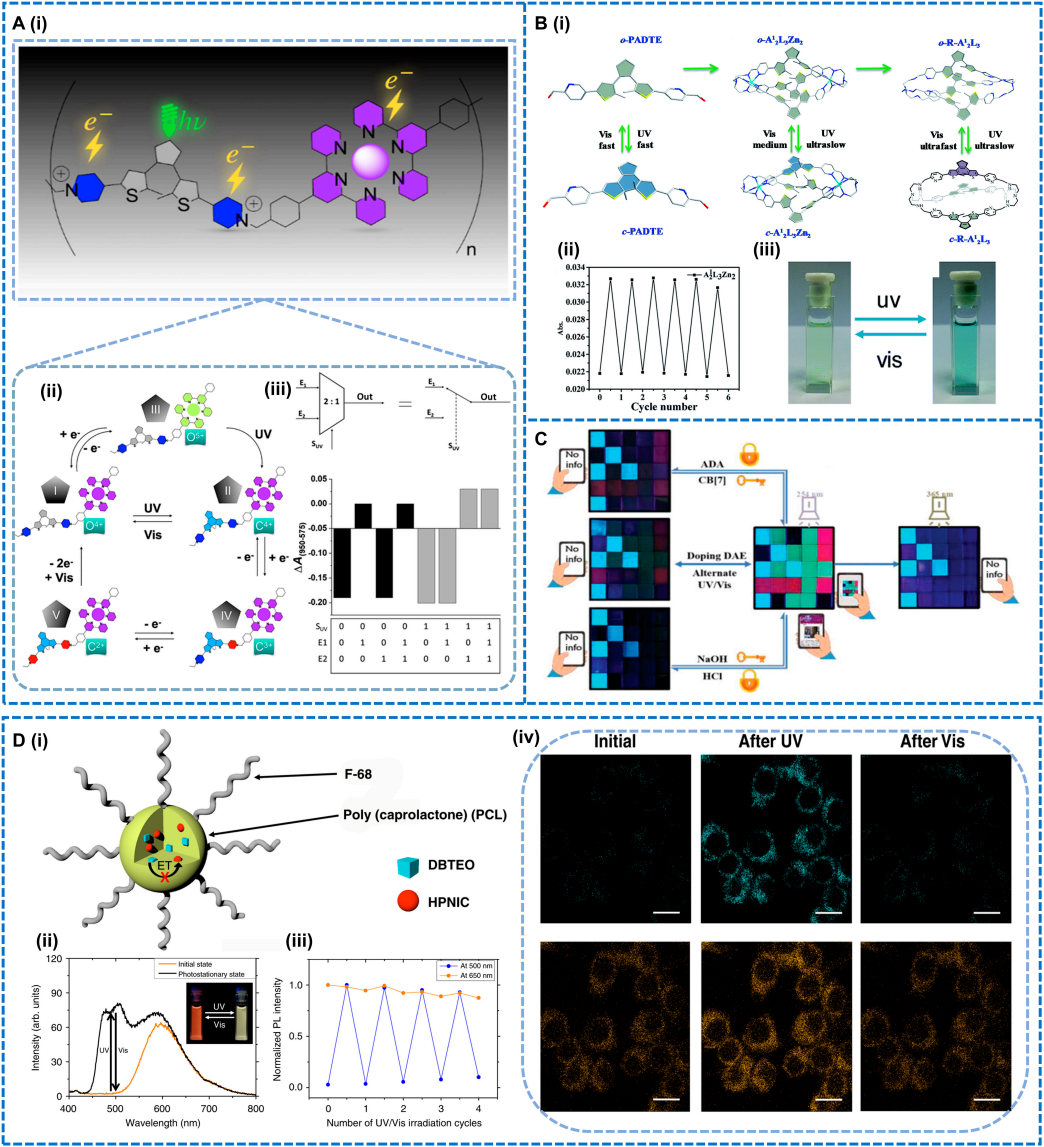
(A-i) Formation and photoinduced isomerization of the coordination polymer. (A-ii) Schematic interconversion of the multistability of the iron metallopolymer switch; for more information about the chemical structure, (A-iii) top: schematic presentation of a 2:1 multiplexer, bottom: performance of poly (FeII-Lo)^4+^ as a 2:1 multiplexer and truth table. Reproduced with permission from [117]. Copyright 2022, American Chemical Society. (B-i) Component self-assembly of photoresponsive A_2_L_3_M_2_ (A = A1 or A2, M = ZnII or CdII) metal-templated cage and UV/vis induced ring-opening/closing behaviors in PADTE. (B-ii) Cycled signals for absorbance at 627 nm during alternate ring-closing/opening processes. (B-iii) Polymer photochromic photos. Reproduced with permission from [118]. Copyright 2020, Royal Society of Chemistry. (C) Combined logic gate application before and after UV multiple-stimulation response: photographs of patterns assembled from multicolored hydrogels on black substrates. Reproduced with permission from [119]. Copyright 2023, John Wiley & Sons Inc. (D-i) Schematic representation of the PCL NPs doped with DBTEO and HPNIC and their photoswitching reaction by UV/visible-light irradiations. (D-ii) Photoluminescence (PL) spectra changes of the PCL NP containing DBTEO and HPNIC upon UV and visible light irradiations. (D-iii) Reversibility test of the NP with alternation of UV and visible light irradiations. (D-iv) Polymer bioimaging applications. Reproduced with permission from [125]. Copyright 2019, Springer Nature.

Later, Chatir et al. [117] synthesized a new iron metal coordination polymer (FeII-L)^4+^ that contained a photochromic DTE (Fig. 10A-i). The polymer could undergo reversible transitions to multiple states under optical and redox stimuli. State I was irradiated with 365-nm UV light to form a fully conjugated state II, and the initial purple solution of state I was quantitatively bleached on complete electrolysis to produce state III. Both processes were fully reversible for 50 cycles. State III underwent closed-loop isomerization upon irradiation at 465 nm. At this point, Fe(III) was reduced to Fe(II), and the color changed to deep purple. After that, state II could be reduced to state IV and then to state V. No degradation was found in this system for more than 50 consecutive redox cycles, and so it could be used as an AND and OR logic gate, as a half adder and multiplexer, providing important numerical processing at the intersection of redox-active ligand complexes and photochromism (Fig. 10A-ii and iii).

To perform complex logic operations using DTEs, multiple photoswitchable DTE components can be integrated into well-defined organic cages, but this has been less well studied. Zhang et al. [118] reported the further metallization of a pure organic cage from a photochromic diphenyl ethylene dipyridyl aldehyde and 2 tripodal triamines (A1 or A2) ligands by self-assembled reduction (Fig. 10B-i). The color of the ligand solution transformed from light yellow (open loop) to blue (closed loop) under 365-nm UV irradiation and returned to light yellow by visible light irradiation with at least 6 photochromic cycles (Fig. 10B-ii and iii). The combination of the photochromic cage with lanthanide upconversion materials allowed multicolor modulation with different irradiation. The photoluminescence colors were also adjusted to achieve the tuning of the photochromic cage in the logic gate model.

With the coassembly of DAE and CB [7], the tunable full-color spectrum was successfully achieved, providing an innovative solution for the application of multilevel logic gates and intelligent multicolor anti-counterfeiting inks (Fig. 10C) [119]. In the future, more photochromic molecules can be doped to expand the wavelength range of the logic gates and make them applicable to a wider spectral region, which will bring more possibilities in the field of optical communication and sensors. This will promote the continuous innovation and application of photochromic DAE logic gates in the field of information processing and optoelectronics.

#### Super-resolution bioimaging

Single-molecule super-resolution microscopy has become a standard imaging tool for in situ visualization of nanostructures in life sciences [120], but the application of this technique to polymers has been less explored [121]. A key bottleneck is the lack of fluorophores and covalent attachment to polymer chains [122]. DAE polymers are ideal for super-resolution imaging because of their remarkable AIE properties. They fluoresce weakly in solution but strongly in the aggregated or solid state, a property that allows them to produce strong fluorescent signals in super-resolution imaging, improving image contrast and resolution. Qiang et al. [123] reported a functional photoswitchable fluorophore prepared by doping DAE into the polymer backbone. The initially colorless fluorophore was isomerized to a bright-yellow state and became highly fluorescent upon irradiation with 375-nm light, and reverted to colorless upon irradiation with 473-nm light.

Dual-color fluorescent NPs also show perfect color-specific light switching for biological imaging and super-resolution microscopy [124]. Kim et al. [125] prepared 2-component NPs consisting of blue fluorescent turned-on DAE and orange fluorescent excited state intramolecular proton transfer dyes (Fig. 10D-i). When 3,3′-(perfluorocyclopent-1-ene-1,2-diyl)bis(2-ethylbenzo[b]thiophene 1,1-dioxide), i.e., DBTEO, was present in the colorless “O” form, the luminescent color of the NPs was orange. After 6 s of UV irradiation, the emission color appeared white due to the appearance of a photochromic blue emission. Reversible color photoswitching of the NPs could be achieved by alternating UV and visible light irradiation (Fig. 10D-ii and iii). The color switching of the NPs could be stably repeated and could therefore be used for biological imaging (Fig. 10D-iv). RAW 264.7 macrophages were treated with the NPs (“O” type) in the off state to investigate their in vitro imaging properties. The C-type blue emission of DBTEO was reversibly switched on and off under repeated UV–visible irradiation, while the orange emission of 3-(1-phenyl-1H-phenanthro[9,10-d]imidazol-2-yl)naphthalen-2-ol (HPNIC) remained almost unchanged, demonstrating that color-specific photoswitching indeed played a role in the intracellular environment due to the high structural integrity of PCL-based NPs. The 365-nm UV irradiation was found to be minimally toxic to cells and could be used for cellular imaging. The color-specific photoswitched NPs were found to be capable of cellular imaging at a high resolution of ~70 nm, rendering them useful for super-resolution imaging techniques in the future. Others have attempted to use bithiophene-based polyethylene polymers to fabricate flexible nonvolatile optical memory thin-film transistor devices with multilevel storage capabilities [126]. By controlling the exposure to light, the DAE molecules can switch between their closed and open forms, changing the conductivity of the transistor and storing different memory states. We anticipate the future application of biphenyl-based polyethylene in flexible electronics and other emerging fields.

## Other Photochromic Polymers

Photochromic polymers have numerous commercial applications beyond academic research. For instance, photochromic materials [127] can be utilized in smart windows, ophthalmic lenses [128], UV printing [129], and so on [130]. The commercialization of photochromic polymers remains a hot topic of current research. In recent years, with the emergence of additive manufacturing, an increasing number of researchers have been focusing on integrating 3D printing with photochromic polymers for commercial utilization. Consequently, this is expected to be a key research focus in the future [131]. Three-dimensional printing, also known as additive manufacturing, is a technology that generates 3D solids by adding material layer by layer through successive stacks of physical layers, which allows the production of 3D structures with a high degree of shape complexity, as opposed to traditional machining techniques that remove material. Common 3D printing technologies include SLA (stereolithography), DLP (digital light processing), LCD (liquid crystal panel), and FDM (fused deposition). There are many reported applications of 3D printing, such as for wearable devices [132], programmable gas sensors [133], structural health monitoring [134], etc.

**Fig. 11.**
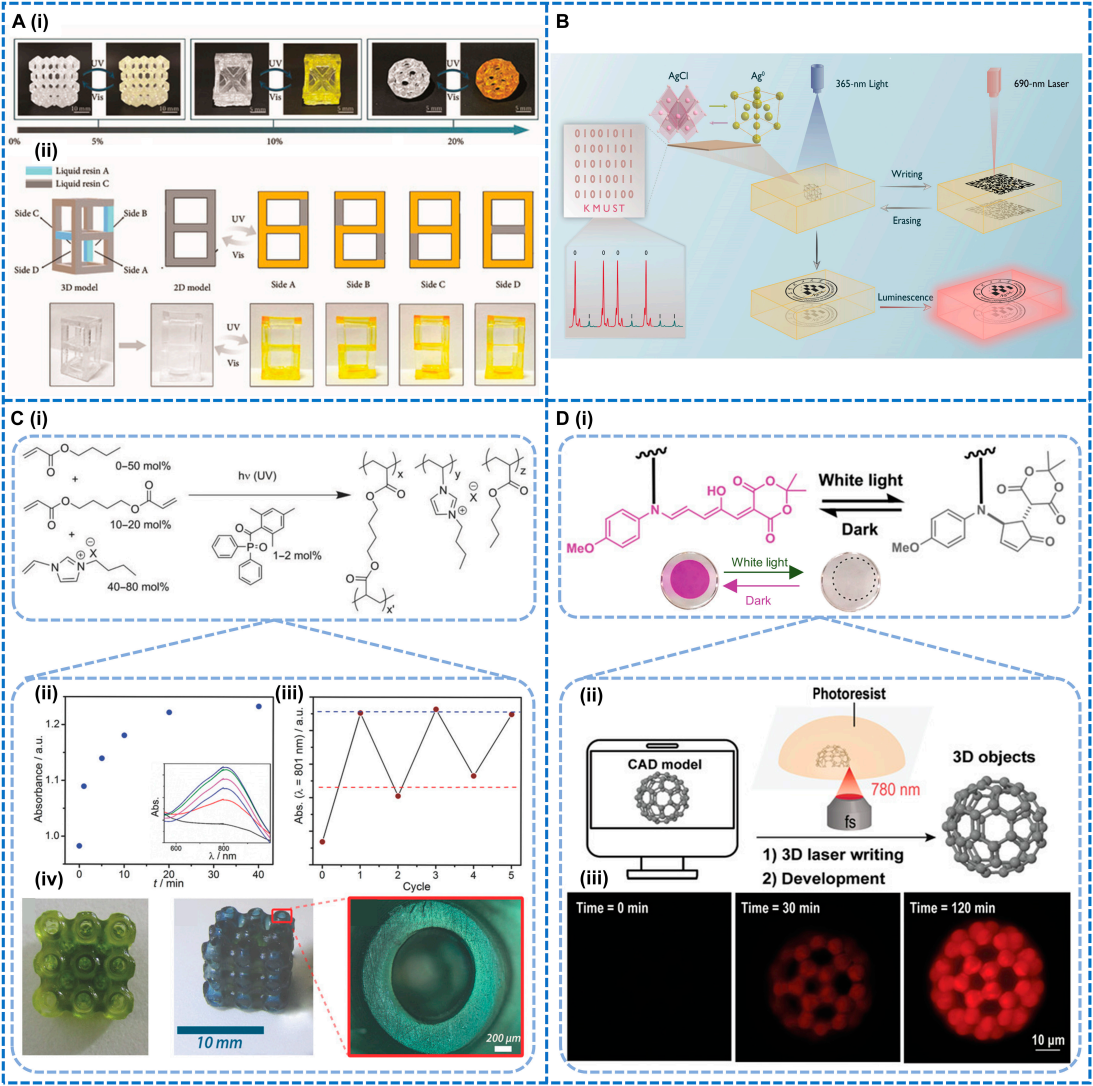
(A-i) Photoresponsive pictures of the printed hollow 3D structures containing different mass fractions of TrPEF_2_-MA. (A-ii) Schematic of the 3D-printed multicomponent framework for the information carrying and encryption. Reproduced with permission from [136]. Copyright 2022, American Association for the Advancement of Science. (B) Schematic illustration of the writing, reading, and erasing of optical information in the transparent Eu-Ag germanium borate glass. Reproduced with permission from [140]. Copyright 2022, American Chemical Society. (C-i) 3D printing formulation composition. (C-ii) Increase in absorption at *λ*_max_ = 801 nm due to photoreduction of the encapsulated [C_10_POM] upon exposure to the projected light over time, and the inset shows the corresponding UV–vis spectra. (C-iii) Information encoded by photoreduction could be erased and reprinted several times, as evidenced by a change in the absorbance intensity at *λ*_max_ = 801 nm upon cycles of photoreduction (encoding) and oxidation (erasure). (C-iv) 3D printed structure accuracy and changes before and after UV irradiation. Reproduced with permission from [137]. Copyright 2018, John Wiley & Sons Inc. (D-i) Schematic depiction of the photoisomerization of a DASA-containing network. (D-ii) Schematic of direct laser writing using a C60-shaped CAD model. (D-iii) Microstructural microscopic images of 3D structures at different time points after laser excitation. Reproduced with permission from [138]. Copyright 2021, John Wiley & Sons Inc.

To date, research on photochromic polymers has focused on the construction of simple 2-dimensional films in polymer matrices by means of doping or gratings [135]. However, with the rapid development of 3D printing technology, such materials with special optical properties are beginning to show great potential in the field of 3D printing. Next, we will explore in depth and review those photochromic polymers for 3D printing. Zhang et al. [136] reported a new light-responsive material, MMA containing triphenyl ethylene (TrPEF_2_), which could be used directly in DLP 3D printing. The synthesis of this triphenylethylene color-changing compound was very simple, with high yields and good reversibility of photochromism. TrPEF_2_ was used in copolymer form to prepare photochromic inks for 3D printing, and the high-resolution 3D objects exhibited excellent solvent/heat resistance, as well as precise and controllable light-responsive properties. The resolution of the prepared precision structures was as high as 3 μm. Moreover, the saturation degree of the 3D structures could be controlled by adjusting the composition ratio of TrPEF_2_. Honeycomb structures (Resin B), modified hollow cubes (Resin C), and porous hollow spheres (Resin D) could be printed by different color-changing inks, and the colors of these structures could respectively change from transparent to yellow, light yellow, and orange. These structures could be switched between transparent and yellow with good repeatability (25 times) under alternating UV and visible light irradiation (Fig. 11A-i). The technology offered a novel approach to the direct design of photoresponsive materials for DLP 3D printing and held promise for future use in adaptive camouflage, information hiding, and information storage (Fig. 11A-ii).

In addition, Wales et al. [137] synthesized a novel amphiphilic organic–inorganic hybrid (C_10_POM) and found it to be soluble in 2 synthetic ionic solutions (hydrophilic and hydrophobic) for the development of smart polymeric materials for DLP 3D printing (Fig. 11C-i). When 3D printing with hydrophobic C_10_POM compositions, a light yellow solid was obtained, indicating that the printed device C_10_POM had not been reduced and was still in the “0” or “off” state. The color of the solid changed to blue after 20 min of UV light exposure and could be cycled 5 times (Fig. 11C-ii), achieving a controlled change in the topology and time of light reduction to the “1” or “on” state (Fig. 11C-iii), enabling reversible photochromic phenomena. The unique light and redox properties of molecularly engineered nanostructured polyoxometalate (POM) could be translated into macroscopic functional devices with reversible photochromic properties (Fig. 11C-iv).

The establishment of common photochromic 3D printing requires UV light, which has a limited and detrimental penetration depth into many materials or skin. On the other hand, donor–acceptor Stenhouse adducts (DASA), a novel visible-light-responsive photoswitch with negative photochromism, can overcome current limitations. For the first time, Ulrich et al. [138] have succeeded in the preparation of a highly efficient thiol-ene photosensitive resin for combination with 2-photon polymerization nanoscale 3D printing technology to fabricate a visible-light-responsive photochromic 3D micro-object (Fig. 11D-i). This photochromic 3D micro-object scanned a focused green laser beam within a functionalized microstructure, enabling dynamic color modulation of the DASA-containing polymer network and its reversible light switching with high spatial resolution. The photochromic 3D micro-objects not only exhibited a high spatial resolution but also allowed the flexible switching between different colors to meet the needs of different applications (Fig. 11D-ii and iii).

Photochromic glasses and multicomponent printing [139] represent potential applications for photochromic polymer 3D printing. Zhao et al. [140] prepared fully photoexcited reversible photochromic germanium borate glasses doped with rare earth ions and AgCl. Under 365-nm laser irradiation, a dark region was formed as the photochromic region, and a reversible photochromic color change from transparent to black was achieved. Optical information could be written inside the lanthanide ion-doped photochromic glass, and the excitation light from a xenon lamp was used to excite the glass so that the optical information could be read. Modulated transmittance or decolorization in the glass could be eliminated using 690-nm laser irradiation. No degradation of luminescence was observed over multiple irradiation cycles, demonstrating the remarkable reproducibility of the Eu-Ag glasses (Fig. 11B). The best prospect for photochromic glass is that it could be used in automobiles, but this has not yet been investigated, and it will be a great breakthrough if DLP 3D-printed photochromic polymers could be used in automobiles in the future.

Affordable 3D printing of photochromic polymers has entered the market in the past and is expected to become increasingly popular shortly. The demand for multimaterial printing is also expected to increase as 3D printing is increasingly used to manufacture functional products or components. The excellent properties of photochromic polymers allow them to be used as anti-counterfeiting media, and it is expected that DLP 3D-printed photochromic polymers will be used in the future for military anti-counterfeiting applications such as aerospace and aviation, as well as in orthopedics, regenerative medicine, and tissue engineering. In conclusion, 3D printing of photochromic polymers holds compelling promise and could be more extensively investigated.

## Summary and Outlook

In this review, we have systematically summarized the recent progress of photochromic polymers in terms of photoisomerization mechanism, polymer classification, and recent applications of photochromic polymers. The applications of 3 photochromic polymers, azobenzene, SP, and diarylethylene, have been studied in detail. Table summarizes the photochromic parameters corresponding to the polymers, including their morphology, color change, number of cycles, and discoloration recovery time. In this paper, azobenzene polymers are classified into 2 categories: BCs and dendritic polymers. Their applications in the fields of photopatterning, drug delivery, and self-healing are also summarized. The use of simpler synthetic azobenzene polymers and their specific applications are still being explored due to their variable structures with complexity and various synthetic methods. Then, the table summarizes the application of SP and diarylvinyl photochromic polymers in security, super-resolution imaging, logical gates, sensors, and so on. This review also summarizes other photochromic polymers, which are characterized by the potential for commercial application in combination with 3D printing. It is expected that in the future, photochromic 3D printing polymers can be applied in sensors, photochromic glass, and potential human tissue structures, thereby improving people’s quality of life.

**Table.** Summary of the photophysical of photochromic polymers discussed in this review. “n” represents “not mentioned in the paper or none of the property”.

		Compound name	Polymer form	*λ*_excitation_/*λ*_recovery_	The absorption maxima	Color changes/ Other changes	Size	Cycle times	Recovery time	Ref.
Azobenzenes	1	ABA	Copolymer solution	366 nm, 8 mW cm^−2^/437 nm, 4 mW cm^−2^		Sol ↔ gel	/	5	3,000 s	[40]
	2	G1-AZO	Fine powder	365 nm, 50 mW cm^−2^/450 nm, 50 mW cm^−2^	450 nm	Orange ↔ dark red	/	10	20 min	[47]
	3	G3-AZO	Fine powder	365 nm, 50 mW cm^−2^/450 nm, 50 mW cm^−2^	450 nm	Yellow ↔ red	/	10	20 min	[47]
	4	G5-AZO	Fine powder	365 nm, 50 mW cm^−2^/450 nm, 50 mW cm^−2^	450 nm	Yellow ↔ red	/	10	20 min	[47]
	6	AZO-LC	Membranes	365 nm/n	450 nm	/	/	21	10 s	[52]
	7	PNB-AZO-100	Spin-coated polymer films	365 nm/450 nm	460 nm	/	/	/	50 s	[53]
	8	PbAzo	Polymer membrane	405 nm, 30 mW cm^−2^/532 nm, 30 mW cm^−2^	485 nm	Yellow ↔ red	/	8	50 s	[54]
	9	PPNPs	Polymeric nanocarriers	365 nm/n	420 nm	/	100 nm	/	/	[61]
	11	P-H	Rotary casting film	365 nm, 1.9 mW cm^–2^/530 nm 2.8 mW cm^–2^	445 nm	Solid ↔ liquid	100 nm	5	5 min	[62]
	12	P-Me	Rotary casting film	365 nm, 1.9 mW cm^–2^/530 nm 2.8 mW cm^–2^	445 nm	Solid ↔ liquid	/	5	5 min	[62]
	13	P1-5K	Polymer membrane	365 nm, 51 mW cm^−2^/470 nm, 9 mW cm^−2^	446 nm	Solid ↔ liquid	/	/	10 min	[63]
Spiropyran	14	PS-b-P4VP	Nanoporous multilayer films	375nm/n	550 nm	Colorless ↔ purple	/	/	15 min	[67]
	15	PS-b-PAA	Nanoporous multilayer films	375nm/n	550 nm	Colorless ↔ purple	/	/	15 min	[67]
	16	P(SpMA-co-MMA)-b-PEG	Nano-membrane	365 nm, 1.2 mW cm^–2^/LED lamp	565 nm	Transparent ↔ purple	/	2	30 min	[71]
	17	SPTS-PSt-b-PEO	Polymeric nanoparticles	365 nm/*λ*>420 nm	590 nm	Colorless ↔ purple	/	7	60–120 min	[72]
	18	PEO-b-PSPA	Vesicles	365 nm, 1.5 mW cm^−2^/530 nm, 1 mW cm^−2^	574 nm	Blue ↔ navy	450 nm	5	10 min	[74]
	19	PEO-b-PSPO	Vesicles	365 nm, 1.5 mW cm^–2^/530 nm, 1 mW cm^–2^	589 nm	/	430 nm	5	10 min	[74]
	20	PEO-b-PSPMA	Micelles	365 nm, 1.5 mW cm^–2^/530 nm, 1 mW cm^–2^	586 nm	/	25 nm	5	10 min	[74]
	21	NIPAM-St-EA	Polymeric nanoparticles	*λ*<400 nm/*λ*> 450 nm	588 nm	Colorless ↔ blue	68 nm	6	20 min	[77]
	22	SPEA	Polymeric nanoparticles	365 nm/ visible light	590 nm	Colorless ↔ purple	90 nm	/	14 min	[11]
	23	MMA-SPEA	Latex particles	365 nm, 6 W m^−2^/LED lamp (8 W m^−2^)	550–560 nm	Colorless ↔ purple	400–900 nm	15	5 min	[21]
	24	MMA-HEMA-SPOH	Polymeric nanoparticles	365 nm/visible light	536–547 nm	Colorless ↔ pink	80 nm	40	5 min	[78]
	25	PBAMMA-SP2	Latex nanoparticles	365 nm/n	558 nm	White ↔ light purple	68 nm	20	5 min	[79]
	26	PMMA-SP	Latex nanoparticles	365 nm/visible light	540 nm	White ↔ purple	57 nm	20	5 min	[79]
	27	C1	Spin-coated film	365 nm/visible light	565 nm	Light yellow ↔ purple	/	100	1 min	[87]
	28	C2	Spin-coated film	365 nm/visible light	565 nm	Light yellow ↔ purple	/	100	2 min	[87]
	29	FPMCNPs-2	Polymeric nanoparticles	365 nm/visible light	532 nm	Colorless ↔ purple	49 nm	/	2 min	[88]
	30	FPMCNPs-4	Polymeric nanoparticles	365 nm/visible light	533 nm	Colorless ↔ light Purple	78 nm	/	2 min	[88]
	31	FPMCNPs-5	Polymeric nanoparticles	365 nm/visible light	526 nm	Colorless ↔ purple	55 nm	/	2 min	[88]
	32	FPMCNPs-8	Polymeric nanoparticles	365 nm/visible light	542 nm	Colorless ↔ light purple	123 nm	/	2 min	[88]
	33	PFPNs	Polymeric nanoparticles	365 nm, 2.8 mW cm^−2^ /525 nm, 1.34 mW cm^−2^	500–550 nm	/	81 nm	7	5 min	[91]
	34	BOSA-SP	Polymer film	365 nm/524 nm	576 nm	Yellow ↔ purple-black	/	5	120 min	[92]
	35	PMMA	Polymeric nanoparticles	365 nm/visible light	549 nm (pH=7)	/	60 nm	25	5 min	[93]
	36	PMMA-co-PHEMA	Polymeric nanoparticles	365 nm/visible light	510 nm	/	55 nm	25	5 min	[93]
	37	PMMA-co-PDMAEMA	Polymeric nanoparticles	365 nm/visible light	520 nm	/	45 nm	25	5 min	[93]
	38	MSP@NF	Nanofiber	365 nm/visible light	578 nm	Colorless ↔ purple	169 ± 39 nm	10	10 min	[95]
	39	MBSP@NF	Nanofiber	365 nm/visible light	584 nm	Colorless ↔ dark purple	203 ± 47 nm	10	10 min	[95]
Diarylethene	40	DPP-1	Conjugated polymer networks	365 nm/>500 nm	656 nm	Pale yellow ↔ grass green	5 μm	3	50 s	[100]
	41	DPP-2	Conjugated polymer networks	365 nm/>500 nm	656 nm	Yellow ↔ olive green	10 μm	3	50 s	[100]
	42	DPP-1/PMMA	Spin-coated film	365 nm/>500 nm	656 nm	Pale yellow ↔ grass green	/	3	50 s	[100]
	43	DPP-2/PMMA	Spin-coated film	365 nm/>500 nm	656 nm	Yellow ↔ olive green	/	3	50 s	[100]
	44	DAE@CB7	Supramolecular complex	365 nm/470 nm	444 nm	/	/	2,560	60 s	[101]
	45	SCP	Printing inks	300 nm/>450 nm	596 nm	Colorless ↔ dark blue	/	4	60 s	[101]
	46	DTE-TPE-AIE	Polymer film	300 nm, 0.14 mW cm^−2^ /620 nm, 0.9 mW cm^−2^	622 nm	Colorless ↔ blue	/	15	Few minutes	[104]
	47	Cu-DTEDBA	Amorphous powder	365 nm/>470 nm	580 nm	Light green ↔ dark blue	/	10	30 min	[105]
	48	PMFP-1	Polymer film	365 nm, 2.36 mW cm^−2^ /525 nm, 30 mW cm^−2^	587 nm	Colorless ↔ purple	/	25	1 min	[110]
	49	SP-3	Spin-coated film	365 nm 2.36 mW cm^−2^ /525 nm, 30 mW cm^−2^	560 nm	Colorless ↔ purple	/	10	180 s	[110]
	50	(FeII-L)^4+^.	Metal coordination polymers	365 nm/660 nm	570 nm	Yellow ↔ purple	/	10	35 min	[117]
	51	PATTE	Organic cage	365 nm/>455 nm	605 nm	Colorless ↔ blue	/	6	10 min	[118]
	52	DBTEO	Small molecule	365 nm/>420 nm	415 nm	/	/	6	15 min	[125]
	53	PCL NPs	Nanoparticle	365 nm/>420 nm	/	/	26.7 ±3.9 nm	4	30 min	[125]
Others	54	TrPEF_2_	Polymer film	365 nm/n	461 nm	Colorless ↔ yellow	3 mm	20	2,520 s	[136]
	55	Resin B	3D Structures	365 nm/n	468 nm	Transparent ↔ light yellow	10 mm	25	40 min	[136]
	56	Resin C	3D Structures	365 nm/n	482 nm	Transparent ↔ bright yellow	5 mm	25	2,310 s	[136]
	57	Resin D	3D Structures	365 nm/n	471 nm	Transparent ↔ orange	5 mm	25	40 min	[136]
	58	C_10_POM	3D Structures	Osram lamp, 210 W	801 nm	Light yellow ↔ blue	10 mm	5	48 h	[137]
	59	Eu-Ag glass	Nanoparticle	365 nm/690 nm, 1.18 W cm^–2^	/	Transparent ↔ black	9.1 nm	50	30 s	[140]

Additionally, the development of photochromic polymers continues to face major challenges, leaving room for valuable research to improve their applicability. One challenge is developing polymers that are easier to synthesize and more responsive to light. This can be achieved by exploring new polymerization methods, such as solution polymerization and interfacial polymerization, to simplify the synthesis process and improve efficiency. The properties of polymers that allow for fast response make it possible to adjust their color and transparency, resulting in rapid changes in images. This makes them easily applicable in optical communication devices, such as optical switches and modulation. In the future, researchers can optimize polymer structures and introduce photosensitive additives to improve their light responsiveness, making them more promising for smart windows or advanced anti-counterfeiting.

Secondly, the development of polymer molecules that are resistant to light fatigue, reversible, and long-lived is critical for flexible electronic device applications. The cycle life of photochromic polymers is a key consideration for wearable device applications such as photochromic lenses, color-changing garments, and color-changing smartwatches. The light transmission of photochromic lenses can be adjusted by light intensity to minimize eye damage, which requires the photochromic polymers to be highly reversible during repeated photochromic processes to ensure that the lenses work stably for long periods of time. At the same time, compounding photochromic polymers with nanomaterials or other functional materials is an effective way to enhance their light stability and cycling performance. In the future, photochromic polymers can also be used in renewable energy devices such as flexible solar cells. By adjusting the light-absorbing properties of polymers, the photoelectric conversion efficiency of solar cells can be improved, realizing the efficient use of energy.

Additionally, given the boom in 3D printing technology in recent years, we are looking forward to the combination of photochromic polymers and 3D printing technology. We expect that researchers will be able to develop photochromic polymer materials that are suitable for various printing techniques and will be used in a wide range of applications such as optical devices and aerospace. In addition, we further expect future 3D printed photochromic polymer structures to be used for structural health monitoring. For example, the use of these polymers to make photochromic sensors could be effective in reducing potential damage to spacecraft by detecting stress–strain during color changes. We also expect to see unique applications of photochromic polymers in the medical field. These polymers can be used not only for color change detection of bacteria and viruses but also for dynamic monitoring of human health by building structures in human tissues using 3D printing technology.

Photochromic polymers are promising, and researchers are working hard to develop better open materials and diverse applications. With the development of cross-disciplines, research on photochromic polymers will also generate more integration and innovation with other fields. For example, the combination with nanotechnology, biotechnology, information technology, and other fields is expected to bring new breakthroughs and application prospects for the development of photochromic polymers. This paper aims to provide basic guidance for the further development of photochromic polymers.
